# EasyPrimer: user-friendly tool for pan-PCR/HRM primers design. Development of an HRM protocol on *wzi* gene for fast *Klebsiella pneumoniae* typing

**DOI:** 10.1038/s41598-020-57742-z

**Published:** 2020-01-28

**Authors:** Matteo Perini, Aurora Piazza, Simona Panelli, Domenico Di Carlo, Marta Corbella, Floriana Gona, Francesca Vailati, Piero Marone, Daniela Maria Cirillo, Claudio Farina, Gianvincenzo Zuccotti, Francesco Comandatore

**Affiliations:** 1 Department of Biomedical and Clinical Sciences “L. Sacco”, Università di Milano, Pediatric Clinical Research Center “Romeo and Enrica Invernizzi”, Milan, 20157 Italy; 2 S.C. Microbiologia e Virologia, Fondazione IRCCS Policlinico San Matteo, Pavia, 27100 Italy; 3 Emerging Bacterial Pathogens Unit, Division of Immunology, Transplantation and Infectious Diseases, IRCCS San Raffaele Scientific Institute, Milan, 20132 Italy; 4 Microbiology Institute, A.S.S.T. “Papa Giovanni XXIII”, Bergamo, 24127 Italy; 5 Department of Pediatrics, V. Buzzi Childrens’ Hospital, Università di Milano, Milan, Italy

**Keywords:** Bacteriology, Clinical microbiology, Bacterial infection

## Abstract

In this work we present EasyPrimer, a user-friendly online tool developed to assist pan-PCR and High Resolution Melting (HRM) primer design. The tool finds the most suitable regions for primer design in a gene alignment and returns a clear graphical representation of their positions on the consensus sequence. EasyPrimer is particularly useful in difficult contexts, e.g. on gene alignments of hundreds of sequences and/or on highly variable genes. HRM analysis is an emerging method for fast and cost saving bacterial typing and an HRM scheme of six primer pairs on five Multi-Locus Sequence Type (MLST) genes is already available for *Klebsiella pneumoniae*. We validated the tool designing a scheme of two HRM primer pairs on the hypervariable gene *wzi* of *Klebsiella pneumoniae* and compared the two schemes. The *wzi* scheme resulted to have a discriminatory power comparable to the HRM MLST scheme, using only one third of primer pairs. Then we successfully used the *wzi* HRM primer scheme to reconstruct a *Klebsiella pneumoniae* nosocomial outbreak in few hours. The use of hypervariable genes reduces the number of HRM primer pairs required for bacterial typing allowing to perform cost saving, large-scale surveillance programs.

## Introduction

Most methods used for the identification and typing of prokaryotes are based on DNA amplification and sequencing. Indeed, the sequence of specific genes can harbour enough information to classify bacteria at species, subspecies or also to a clonal level. For instance, Multi-Locus Sequence Typing (MLST) is one of the most used methods for bacterial typing and it is based on the amplification and sequencing of few housekeeping genes^1^. During the last ten years, the analysis of the entire bacterial genome by Whole Genome Sequencing (WGS) approach revolutionized the field, drastically increasing the typing precision^1^.

The reconstruction of nosocomial outbreaks is one of the most important clinical applications of bacterial typing. A nosocomial outbreak occurs when the number of patients infected by a pathogen increases above the expected in a limited time^2^. In these situations, it is fundamental to determine the clonality of bacteria causing disease in the patients to define the proper strategy to handle the emergency. Pulsed-Field Gel Electrophoresis (PFGE), MLST and WGS are the most frequently applied molecular methods in outbreak investigation^1^.

During a nosocomial outbreak, clinicians need bacterial typing information in the shortest time possible. Despite the high potential of WGS in outbreak reconstruction, the sequencing of a complete genome requires two to four working days, introducing an important time lag. Similarly, PFGE typing requires five days and also MLST needs few days. During the last years, the High Resolution Melting (HRM) assay has emerged as a low-cost and fast method for bacterial typing, particularly promising for epidemiological applications^3–6^. HRM is a single-step procedure for the discrimination of sequence variants on the basis of their melting temperature. This method allows to perform bacterial typing in less than five hours^7^.

To develop a novel HRM-based typing procedure, it is necessary to: i) select one or more core genes; ii) design a primer pair in conserved regions flanking a gene portion where the melting temperature varies among the strains.

Andersson and colleagues have developed the “MinimumSNP” tool^8^, which identifies, in a gene alignment, the variable positions that can lead to a melting temperature change (called informative SNPs). MinimumSNP identifies single informative positions, not necessarily grouped in a single portion of the gene. In other words, it does not indicate which regions are more suitable for primer design: two low-variable regions neighbouring a SNP-rich informative stretch. Thus, the user of MinimumSNP has to choose one (or few) SNPs and then design primers around it (or around them).

Herein, we present EasyPrimer, a web-based tool for the identification of the gene regions suitable for primer design to perform HRM studies and any kind of pan-PCR experiments. Moreover, we validated EasyPrimer by designing HRM primers for the discrimination of clinical isolates of *Klebsiella pneumoniae*, an important opportunistic pathogen frequently cause of infections in humans and animals^9^.

This article was submitted to an online preprint archive^10^.

## Results

### EasyPrimer: a tool for primers design

EasyPrimer is a user-friendly open-source tool developed to assists primer design in difficult contexts, e.g. on an alignment of hundreds of sequences and/or on hypervariable genes. The tool uses as input a nucleotide multi-fasta file and identifies the best regions for primer design: two low variable regions flanking a highly variable one. The on-line and the stand-alone versions of the tool are freely available at https://skynet.unimi.it/index.php/tools/easyprimer.

### Primers design

**Figure 1:**
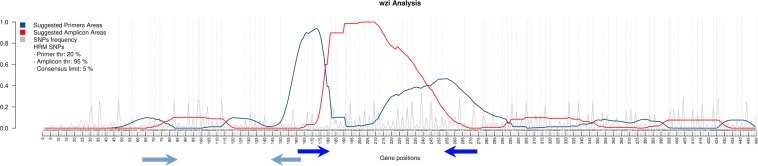
EasyPrimer output for the *wzi gene* (563 alleles on https://bigsdb.pasteur.fr). The consensus sequence calculated from the gene alignment is reported on the x-axis. Residues under the peaks of the blue curve are highly conserved and thus suitable for primer design. Conversely the red curve increases over the highly variable regions suggested to be amplified. The grey peaks represent all the Single Nucleotide Polymorphisms (SNPs) with their own frequency. The dotted lines are used to highlight the “HRM-detectable” SNPs, i.e. the ones causing a change in the GC content. The blue arrows were manually added to show the positions of the two primer pairs designed on *wzi* in this work.

We downloaded *pgi*, *gapA* and *wzi* gene sequences from BigsDB database (https://bigsdb.pasteur.fr) and we run EasyPrimer to identify the best regions for primer design. The EasyPrimer output for the *wzi* gene is reported in Fig. 1, while the outputs relative to *pgi* and *gapA* genes are reported in Supplementary Figs. S1 and S2, respectively. Then we designed a total of four novel primer pairs: one for *pgi*, one for *gapA* and two for *wzi* (reported in Table 1).

**Table 1:** Primer pairs used in this work.

Gene name	Primer name	Primer pair name - in this work	Primer scheme name	Sequence (5′ - 3′)	Amplicon length (bp)	Reference
*infB*	*infB*729-F	*infB-1*	MLST6, MLST8	CCTGCCGGAAGAGTGG	50	Andersson *et al*. 2012
	*infB*729-R		MLST6, MLST8	TCGCGGAAACGTGGAC		Andersson *et al*. 2012
*mdh*	*mdh*1197-F	*mdh-1*	MLST6, MLST8	ATTGCCGACCTGACTAAACG	58	Andersson *et al*. 2012
	*mdh*1197 R		MLST6, MLST8	CTTTCGCTTCCACGACTTC		Andersson *et al*. 2012
*phoE*	*phoE*2013-F	*phoE-1*	MLST6, MLST8	GAAGGGGTGGGGAGTGA	78	Andersson *et al*. 2012
	*phoE*2013-R		MLST6, MLST8	GGCGTTCATGTTTTTGTTGA		Andersson *et al*. 2012
*rpoB*	*rpoB*2227-F	*rpoB-1*	MLST6, MLST8	TGATTAACTCCCTGTCCGTGT	132	Andersson *et al*. 2012
	*rpoB*2227-R		MLST6, MLST8	CGTAGTTGCCTTCTTCGATAGC		Andersson *et al*. 2012
*tonB*	*tonB*2693-F	*tonB-1*	MLST6, MLST8	GTTGAACCCGAACCTGAGC	101	Andersson *et al*. 2012
	*tonB*2693-R		MLST6, MLST8	GGTTTGGGCTTCGGCTTA		Andersson *et al*. 2012
*tonB*	*tonB*2886-F	*tonB-2*	MLST6, MLST8	AAAAGGTTGAACAGCCGAAG	120	Andersson *et al*. 2012
	*tonB*2886-R		MLST6, MLST8	CCGCTGCTGTCGAGGT		Andersson *et al*. 2012
*gapA*	*gapA*_F	*gapA-1*	MLST8	AAAGTCGTTCTGACTGGC	95	This work
	*gapA*_R		MLST8	TTRAAACGATGTCCTGGC		This work
*pgi*	*pgi*_F	*pgi-1*	MLST8	CCAAAATGGTACCGTGCGATT	156	This work
	*pgi*_R		MLST8	CCTGATCGCGRTATTCCTGCT		This work
*wzi*	*wzi*3_F	*wzi-3*	wzi	GCTTAYGCRGCYGGGTTAGTRGT	114	This work
	*wzi*3_R		wzi	GGCCASGTCGACARGCTCAG		This work
*wzi*	*wzi*4_F	*wzi-4*	wzi	GCCGCTRAGYCAGGAAGAGAT	101	This work
	*wzi*4_R		wzi	GACTGTCWGCBTTRAAAGCSGA		This work

### High-resolution melting analysis

In this work we considered three clinical strains collections:

- the “background” collection, which includes 17 *K. pneumoniae* strains belonging to 17 different Sequence Types (STs);
- the “outbreak” collection, which includes 11 *K. pneumoniae* strains isolated during a nosocomial outbreak;
- the “validation” collection that includes 54 *K. pneumoniae* strains belonging to six of the most epidemiologically relevant STs (i.e. ST258, ST512, ST11, ST101, ST15 and ST307)^11,12^.

The strains of the background and outbreak collections were analysed using all the ten primer pairs listed in Table 1. The strains of the validation collection were subjected to HRM experiments using only the two primer pairs designed on *wzi* gene (*wzi-3* and *wzi-4* primer pairs). Four out of the ten primer pairs were newly designed in this work (see above), while the remaining six were already available in literature^7^. The obtained melting temperatures (“Tm”) of the three HRM replicates and their relative average temperature (“aTm”) values are reported in Supplementary Table S1.

### Primer pairs and schemes comparison

**Figure 2:**
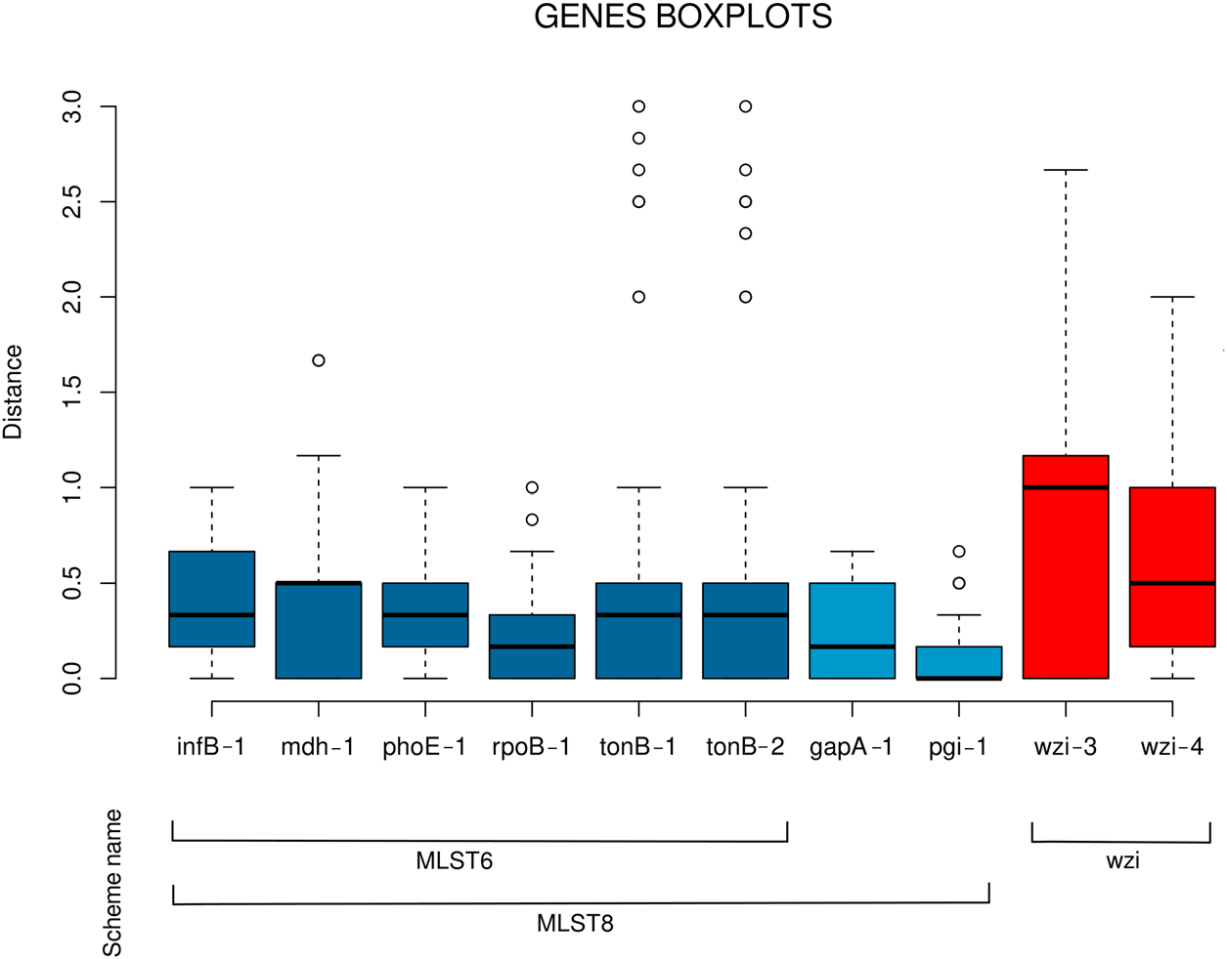
Distribution of the average melting temperature differences among the 17 *Klebsiella pneumoniae* strains for the ten primer pairs. Boxes are the 25th and 75th quartiles divided by the medians, whiskers are 1.5x the interquartile ranges and dots are outliers. The lines in the bottom show the composition of the three primer schemes used in this work.

For each of the ten primer pairs we calculated the strain distance matrix among the background collection strains based on the aTm values (see Methods). The calculated aTm distances ranged from zero to three degrees, and the median distances varied among the genes (as shown in Fig. 2). In particular, the two *wzi* primer pairs showed median distance values significantly higher than those obtained for many of the other primer pairs (see Supplementary Table S2 for details).

We also compared the aTm distance matrices of the following schemes:

- “MLST6”: which includes the HRM-MLST primer pairs already present in literature;
- “MLST8”: which includes the MLST6 primer pairs and the two newly designed;
- “wzi”: which includes the two primer pairs designed for the *wzi* gene.

The median pairwise distance did not significantly change among the three schemes (Wilcoxon test with Holm post-hoc correction, p-value > 0.05) and the relative boxplot graphs are reported in Supplementary Fig. S3.

**Figure 3:**
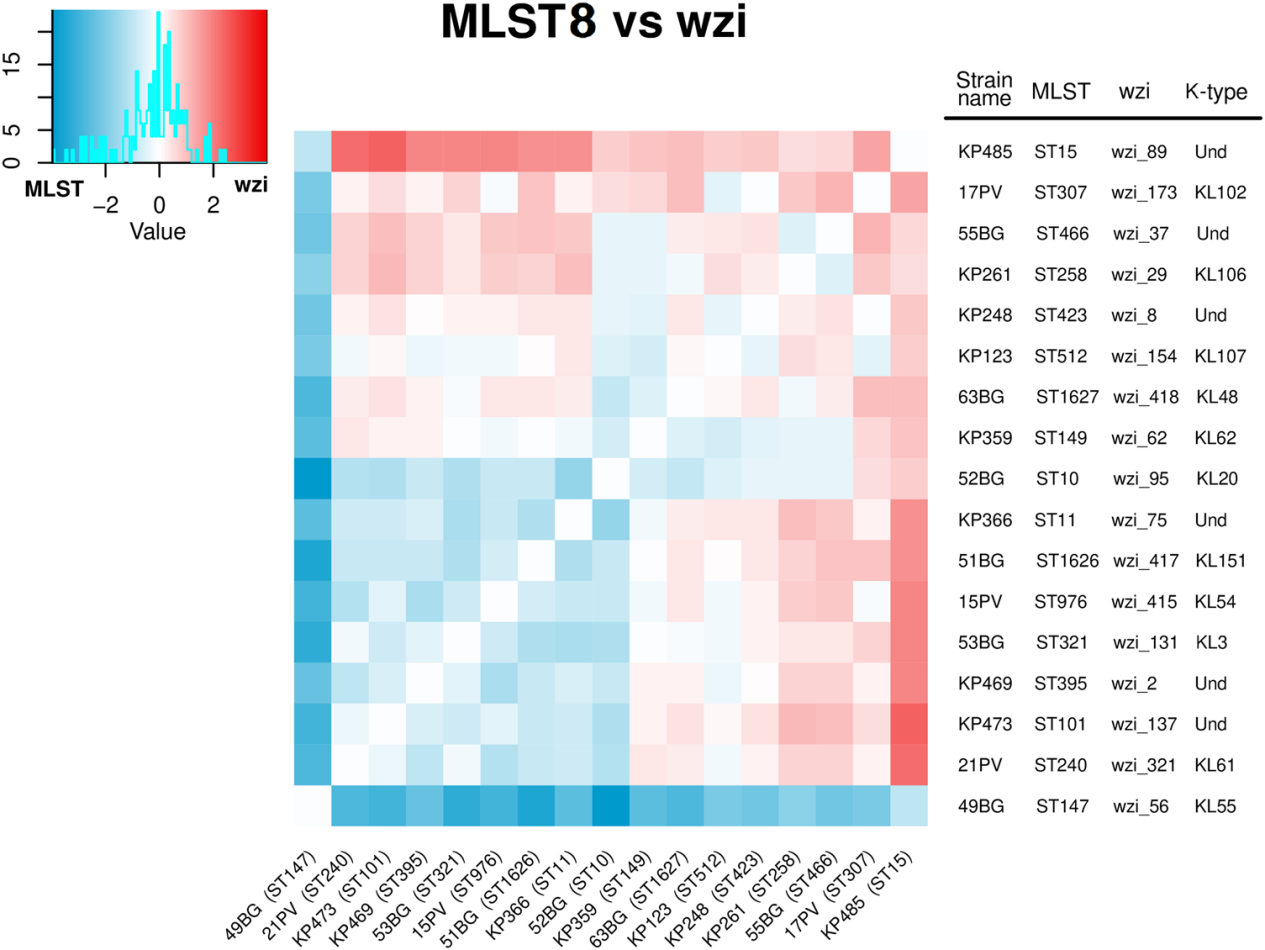
Arithmetic difference between the average melting temperature distance matrices computed among the 17 *Klebsiella pneumoniae* strains (selected to belong to 17 different STs) using the MLST8 scheme (eight primer pairs on seven genes) and wzi scheme (two primer pairon one gene). The heatmap colours range from blue to white to red: if the temperature distance between two strains is greater for the MLST8 than the wzi scheme the relative position on the heatmap is coloured in blue, otherwise in red.

Furthermore, we compared the aTm distance matrices of wzi and MLST8 schemes for each strain pair of the background collection, subtracting the two matrices (see Fig. 3). We found that, among all the 136 possible strain pairs, 66 (48.5%) showed a higher distance for wzi scheme than MLST8 scheme. More in detail, the ST258 *wzi_29* (also known as ST258 Clade 1^13^) was better discriminated from the ST512 *wzi_154* (which is part of the ST258 Clade 2^14^) by wzi scheme than MLST8 (see Fig. 3). ST307 was better or equally discriminated from all the other strains by wzi scheme than MLST8 (except for the ST512 and ST147 strains). Similarly, ST15 (*wzi_89*) strain was better discriminated from all the background strains by wzi scheme than MLST8, apart from the ST147 strain (see Fig. 3). Furthermore, the ST101 strain was better discriminated by wzi than MLST8 scheme from ST258 (*wzi_29*), ST307, ST512 and ST15 (*wzi_89*). ST11 (*wzi_75*) was better discriminated by wzi scheme from ST15 (*wzi_89*), ST307, ST258 and ST512. Conversely, ST11 (*wzi_75*) and ST101 were better discriminated by MLST8 than wzi. Lastly, ST147 was the only ST better discriminated by MLST8 scheme than wzi for all the strains pairs (see Fig. 3).

### Whole genome sequencing-based strain typing

A total of 82 *K. pneumoniae* strains have been subjected to WGS-based typing in this work.

24 out of 82 strains have been previously subjected to NGS sequencing as part of two published works:

- 12 from Gaiarsa and colleagues^14^ (10/12 for the background and 2/12 for the validation collection).
- 12 from Gona and colleagues^15^ (8/12 included in the background collection and 4/12 included in the validation collection).

For these strains, the NGS sequences were retrieved from public database.

For the remaining 58 out of 82 *K. pneumoniae* strains, the reads and genomic sequences were obtained in this work (see Supplementary Table S3):

- 11/58 strains of the outbreak collection (all from Papa Giovanni XXIII hospital).
- 47/58 strains of the validation collection (45/47 from San Raffaele hospital and 2/47 from Papa Giovanni XXIII hospital).

The *wzi* allele, the ST, the K-type as well as the accession numbers of these 82 *K. pneumoniae* strains are reported in Supplementary Table S3.

### WGS-based outbreak reconstruction

Ten out of the 11 outbreak isolates belonged to the ST512 while the isolate “BG-Kpn-22–18” belonged to the ST307 (see Supplementary Table S3). An alignment of 66 core-SNPs was obtained from the 11 outbreak strains. The relative Maximum Likelihood phylogenetic tree is reported in Supplementary Fig. S4. The ST512 strains have SNP distances ranging from zero to four SNPs. Conversely, the SNP distances among the ST512 strains and the ST307 strain ranged from 63 to 66 SNPs.

### HRM-based outbreak reconstruction

**Figure 4:**
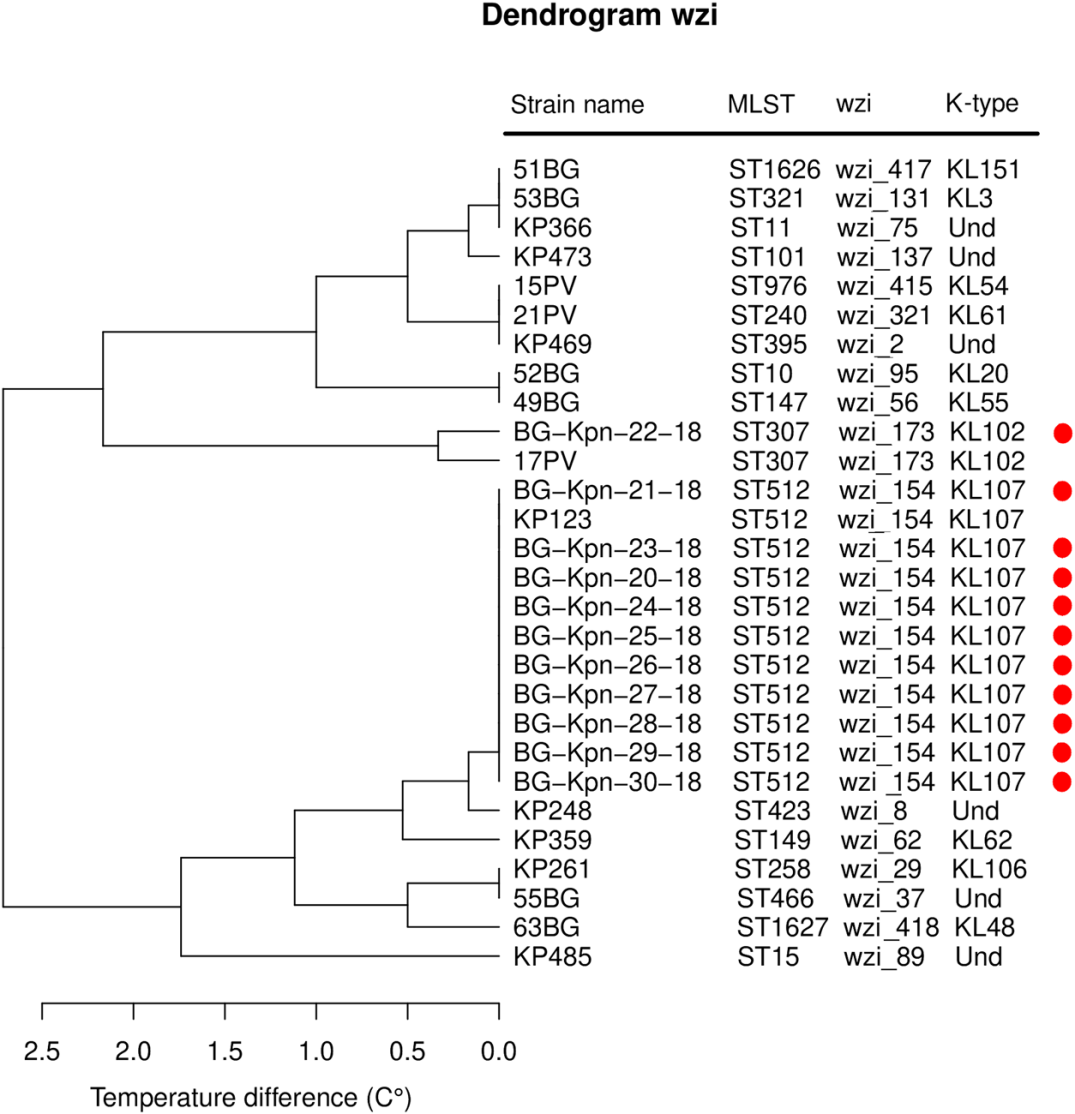
Dendrogram of the hierarchical clustering analysis on the average temperature distance matrix obtained using the wzi primer scheme. The 17 “background” strains belonging to 17 different MLSTs are written in black, while the 11 strains isolated during the nosocomial outbreak are highlighted by a red dot.

The three dendrograms obtained by hierarchical clustering on the aTms strain distances for the schemes MLST6, MLST8 and wzi are reported in Supplementary Figs. S5 and S6 and Fig. 4, respectively. All the schemes correctly discriminated the outbreak ST512 strains from the ST307 one. Notably, only the wzi scheme correctly clustered the outbreak strains with the background strain of the same ST.

### Repeatability of the *wzi* HRM protocol

To validate the repeatability of the *wzi* HRM typing protocol, we included in the analysis 54 additional strains belonging to the most epidemiologically relevant clades (ST258 Clade 1, ST258 Clade 2, ST307, ST101, ST11 and ST15)^11,12^, for a total of 82 strains (see Supplementary Table S3).

For each of these clades, the aTms obtained for *wzi-3* and *wzi-4* primer pairs varied in a range <  = 0.5 °C, corresponding to the sensitivity of the machine used for the HRM experiments (see Table 2).

**Table 2:** Ranges of *wzi-3* and *wzi-4* melting temperatures of the most epidemiologically relevant clades and Sequence Types.

Clade	MLST profile	*wzi* allele	Min-Max *wzi-3* T° (°C)	Min-Max *wzi-4* T° (°C)	Range *wzi-3* T° (°C)	Range *wzi-4* T° (°C)	# of strains
ST258 Clade 2	ST512	*wzi_154*	83.50–84.00	83.33–83.50	0.50	0.17	23
	ST258	*wzi_154*	83.83–83.83	83.50–83.50	0	0	3
ST258 Clade 1	ST258	*wzi_29*	83.50–84.00	84.33–84.67	0.50	0.34	8
ST307	ST307	*wzi_173*	84.00–84.50	82.33–82.83	0.50	0.50	19
ST101	ST101	*wzi_137*	84.83–85.00	83.33–83.83	0.17	0.50	12
ST11	ST11	*wzi_24*	85.00–85.00	83.50–83.50	0	0	2
ST15	ST15	*wzi_24*	85.00–85.17	83.83–83.83	0.17	0	2
	ST15	*wzi_89*	82.33–82.33	84.00–84.00	0	0	1

**Figure 5:**
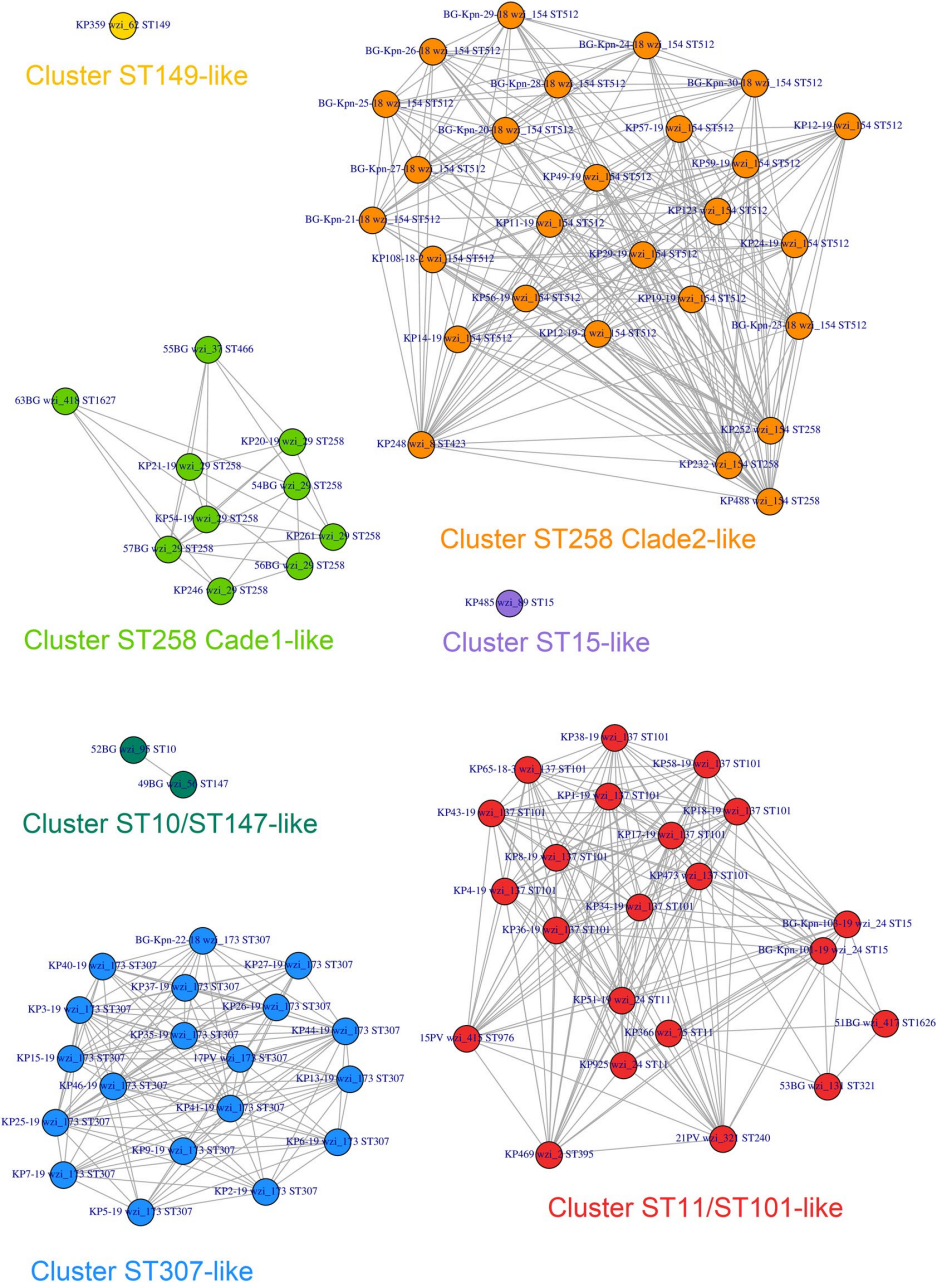
Strain-to-strain network of the 82 isolates analysed, generated on the basis of *wzi-3* and *wzi-4* HRM melting temperatures. Two strains were connected if both *wzi-3* and *wzi-4* gave difference in melting temperature < 0.5 °C. Clusters were identified as separated sub-networks on the strain-to-strain network and they were named from the major Sequence Type they include.

**Figure 6:**
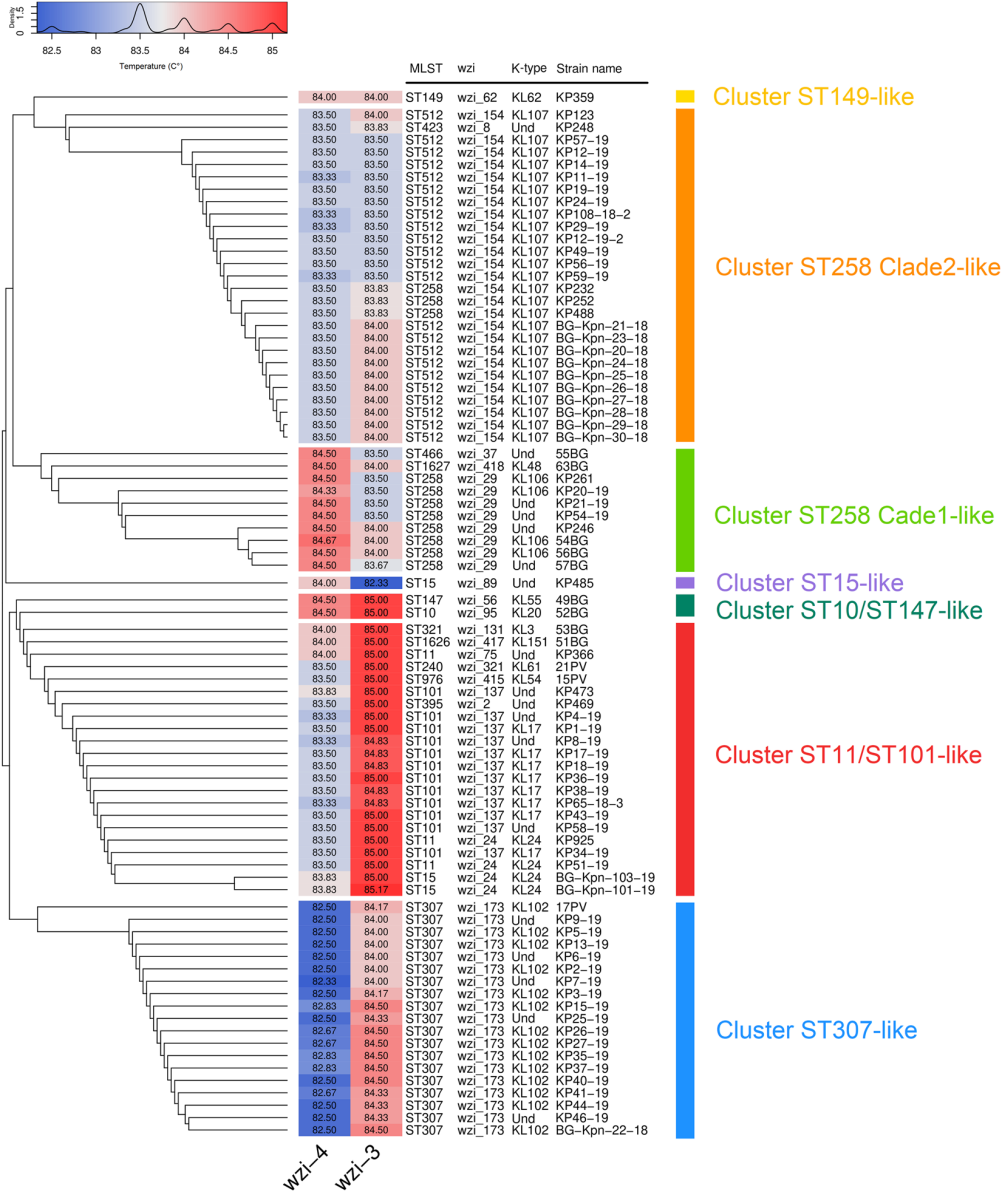
On the left the dendrogram obtained from the strain-to-strain network reported in Fig. 5. In the middle, the average melting temperatures of *wzi-3* and *wzi-4* primer pairs, the Sequence Type, the *wzi* allele and the K-type of the 82 isolates analysed. On the right, the names of the clusters identified by network analysis, corresponding to the clusters in Fig. 5.

Clustering analysis on *wzi-3* and *wzi-4* melting temperatures grouped the strains in seven clusters (see Figs. 5 and 6). Among the most epidemiologically relevant clades, all the strains of the ST258 Clade 1 (ST258 *wzi_29*), ST258 Clade 2 (ST258 *wzi_154* and ST512 *wzi_154*) and ST307 were correctly clustered; while ST11 and ST101 strains fell in the same cluster. The three ST15 strains fell in two different clusters coherently to their *wzi* alleles: the one harbouring *wzi_89* clustered alone, while the other two strains, both harbouring *wzi_24*, fell in the ST11/ST101 cluster (see Figs. 5 and 6).

## Discussion

High Resolution Melting (HRM) is a real-time PCR analysis for the detection of mutations and polymorphisms^3,4^, also applicable for fast bacterial typing in hospital surveillance and real-time nosocomial outbreak detection^5^. Several works applied HRM to bacterial typing, exploiting Multi Locus Sequence Type (MLST) genes^7,16,17^ which have been considered the gold standard genes for bacterial typing for almost 20 years^18^. These genes have been selected to be housekeeping therefore they display low variability. In this work we show that it is possible to increase HRM discriminatory power using hypervariable genes.

On the other hand, the identification of the regions suitable for primer design can be challenging when the number of aligned sequences is high or when the gene is hypervariable. Thus, we developed EasyPrimer, a tool for the identification of the best regions for primer design for HRM analysis and, more in general, for any kind of pan-PCR study. EasyPrimer shows, with an easy-to-read graphical output, which are the best regions for primer design: two conserved regions flanking a highly variable one. The on-line and the stand-alone versions of the tool are freely available at https://skynet.unimi.it/index.php/tools/.

We validated the tool designing HRM primers for the nosocomial pathogen *Klebsiella pneumoniae*. A scheme including six HRM primer pairs for five out of the seven *K. pneumoniae* MLST genes was already available in literature^7^ (MLST6 scheme). Thus, we used EasyPrimer to design the primers for the remaining two MLST genes (*pgi* and *gapA*), obtaining a larger scheme with eight primer pairs (MLST8 scheme). Furthermore, we designed two HRM primer pairs for the hypervariable capsular gene *wzi* (wzi scheme). We tested the discriminatory power of these schemes on 17 *K. pneumoniae* strains belonging to 17 different STs (background collection) and we used the HRM approach to study an outbreak occurred in an Italian hospital.

Notably, most of the epidemiologically relevant *K. pneumoniae* clades (and/or STs) emerged after large recombination events that involved the capsule locus (which includes *wzi* gene), often leading to K-type change. For this reason, the emergence of a novel clade/ST is often associated to *wzi* allele change^19^. This makes *wzi* gene particularly suitable for *K. pneumoniae* typing. Our analyses on the background collection showed a good discriminatory power for both the MLST-based and *wzi*-based HRM assays: both schemes successfully discriminated most of the analysed strains. Wzi scheme discriminated better than MLST8 scheme all the highly epidemiologically relevant clades (ST258 Clade 1, ST258 Clade 2, ST307, ST11, ST101 and ST15), except for the pairs ST258 Clade 2 - ST307 and ST11 - ST101 (see Fig. 3). Nonetheless, wzi scheme is able to discriminate the ST258 Clade 2 from ST307 as well (see Fig. 4). Conversely, *wzi* scheme does not discriminate ST11 from ST101 (see Figs. 5 and 6). The latter may represent a minor flaw of the *wzi* HRM protocol as the two STs are mostly isolated in geographically distant areas of the globe (namely: ST11 in Asia^20^, ST101 in Europe-Africa^21^).

Clustering analysis on the 82 strains, including multiple strains from the same clade (see Materials), allowed to evaluate with more precision the discriminatory power of the *wzi* HRM protocol. The analysis clearly showed that the protocol is able to discriminate five of the six most epidemiologically relevant *K. pneumoniae* clades, discriminating ST258 Clade 1, ST258 Clade 2, ST307, ST11/ST101 and ST15 (see Figs. 5 and 6). In our dataset we found strains of the ST15 and ST11 harbouring different *wzi* alleles. This is not surprising, considering that the capsule locus (which contain the *wzi* gene) is a recombinational hotspot in ST11^19^. Wzi scheme discriminated among the different ST15 strains present in the collections, according to the different *wzi* alleles they harbour (see Figs. 5 and 6). This highlights the benefits of using hypervariable genes instead of MLST genes in typing methods: e.g. *wzi* HRM protocol can rule out an ST15 outbreak of strains harbouring different *wzi* alleles, any MLST-based protocol cannot.

As stated above, *K. pneumoniae* clades are often associated to specific *wzi* alleles and K-types. Despite the *wzi* HRM protocol was designed on *wzi* gene, it correctly discriminates most of the epidemiological relevant clades. Furthermore, we found that every K-types correspond to a specific *wzi* allele (see Supplementary Table S3).

Moreover, the analysis of the 82 strains clearly showed that the *wzi* HRM protocol is highly repeatable: *wzi-3* and *wzi-4* aTms ranged within 0.5 °C (the machine sensitivity) among the strains of the same clade (see Table 2). For this study, dozens of independent HRM experiments have been performed in different months by two different operators (M.P. and A.P.). The observed stability of HRM aTms for each clade clearly shows that the results of *wzi* HRM protocol are portable. This makes the method suitable for studies involving several isolates, such as large hospital surveillance programs.

Additionally, we want to highlight that the observed HRM discriminatory power was obtained using a BioRad CFX Connect real-time PCR instrument (BioRad, Hercules, California): a machine not specifically designed for HRM experiments but for real-time PCR, with a melting temperature sensitivity of 0.5 °C (i.e. a lower sensitivity compared to HRM machines).

We applied the wzi scheme to the reconstruction of a nosocomial outbreak occurred in an Italian hospital. During the outbreak, 11 patients resulted colonized or infected by *K. pneumoniae* and the WGS typing revealed that the isolates belonged to two different clones. These clones were identified on the basis of core SNP distance (SNP distance < 5) and MLST profile (one isolate belongs to the ST307 and ten isolates to the ST512). As shown in Fig. 4, the wzi scheme not only correctly discriminated the outbreak isolates of the two clones but it also clustered them with the background isolates of the corresponding ST profile.

During the last years, WGS has revolutionized clinical microbiology, allowing the precise description of bacterial genomic features in few days (including the presence of resistance and/or virulence factors). Despite this, its application during real-time outbreak reconstructions still shows some limits: the time required to be completed, the cost and the necessity of qualified personnel for library preparation, bioinformatic analyses and results interpretation. Indeed, the complete sequencing of a bacterial strain genome costs at least ~100 euros (using an Illumina MiSeq machine) and requires one or two days for library preparation and 5–36 hours for sequencing. During the first days of a nosocomial outbreak the number of cases still increases slowly. In this time frame, it is crucial to quickly understand if the bacterial strains are genetically related, and if the clone is spreading in the nosocomial environment. In this situation HRM is a “first-line” typing technology to figure out when an outbreak is starting. Indeed, HRM is less precise than WGS but it can be reliable for a fast, preliminary bacterial typing, fundamental in the first days of a nosocomial outbreak. If the outbreak is identified, WGS could be used to further investigate the transmission dynamics. HRM assay represents a fast, simple and time/cost saving approach for bacterial typing, allowing to analyse several bacterial samples per days. Furthermore, this technique does not require advanced skills in molecular biology and the results can be analysed without the use of any specific software. This method can be useful also in veterinary and dairy farming settings: *K. pneumoniae* is a relevant veterinary pathogen and one of the most frequent cause of mastitis in dairy cattle^9^.

We found that the discriminatory power of an HRM scheme does not strictly depend on the number of genes but also on their genetic variability. Indeed, comparing the MLST6 and MLST8 schemes, we found that the median distance among the strains did not change significantly. Wzi scheme contains two primer pairs and this reduces drastically the amount of time and costs required for typing. For instance, using only two primer pairs on a 96-well PCR plate, it is possible to type 15 isolates per run (five hours, including DNA extraction, HRM run and analysis of results) with a cost of ~5 euros each. This makes the HRM a feasible method for real-time surveillance and for a preliminary typing step in large epidemiological studies. Lately, Multi-Drug Resistance (MDR) *K. pneumoniae* strains have become a major burden for public health worldwide. Despite WGS represents an important tool for precise bacterial typing, it remains too demanding for developing countries healthcare systems. Low cost and simple protocols, as the *wzi* HRM typing proposed here, represent a real opportunity for surveillance programs.

The use of hypervariable genes in HRM-based bacterial typing, such as *wzi* in *K. pneumoniae*, can drastically increase the discriminatory power of the method. With the large number of genomes available in databases, it is now possible to find the most variable genes for a species. Unfortunately, it is not easy to identify the best regions to design primers in such hypervariable genes, particularly when hundreds of different alleles are available. EasyPrimer can represent a useful tool to overcome this limit.

## Methods

### Isolates collections

We considered three strain collections: the background, the outbreak and the validation collections. The background collection includes 17 strains belonging to 17 different STs retrieved from two previously WGS typed bacterial collections: nine strains from Gaiarsa and colleagues^14 and eight strains form Gona and colleagues15^ (for details see Supplementary Table S3). The outbreak collection includes 11 *K. pneumoniae* isolates gathered during a 16 days nosocomial outbreak occurred in April 2018, in the Papa Giovanni XXIII hospital (Bergamo) (For details see Supplementary Tables S3 and S4). The validation collection includes 54 *K. pneumoniae* isolates belonging to six of the most epidemiologically relevant clades^11,12^:

- 17 strains belonging to ST307.
- 15 strains belonging to ST258 Clade 2, including ST258 *wzi_154* and ST512 *wzi_154*.
- seven strains belonging to ST258 Clade 1 (ST258 *wzi_29*).
- 11 strains belonging to ST101.
- two strains belonging to ST11.
- two strains belonging to ST15.

45/54 strains were isolated at San Raffaele hospital (Milan), 2/54 strains were isolated at Papa Giovanni XXIII hospital (Bergamo), 4/54 retrieved from the *K. pneumoniae* collection of Gona and colleagues^15^ 3/54 retrieved from the *K. pneumoniae* collection of Gaiarsa and colleagues^14^.

Neither ethics committee approval, nor informed consent were required as all collected data are fully anonymized, there was no contact with patients and/or their families and no interventions or changes to treatment and management were made, in accordance with institutional guidelines.

### DNA extraction and whole-genome sequencing

The genomic DNA of the 45 strains isolated from San Raffaele hospital (Milan) were extracted using Maxwell 16 Cell DNA purification kit. The extracted DNA was sequenced using the NextSeq. 500 platform with 2 × 150 bp paired-ends runs, after Nextera XT library preparation.

The genomic DNA of the 13 strains isolated from Papa Giovanni XXIII hospital (Bergamo) was extracted using the DNeasy blood and tissue kit (Qiagen, Hilden, Germany) following the manufacturer’s instructions. The extracted DNA was sequenced using the Illumina Miseq platform with a 2 × 250 bp paired-end run, after Nextera XT library preparation.

The genomic DNA of the 12 strains previously sequenced by Gaiarsa and colleagues^14^ were extracted using QIAsymphony Virus/Pathogen minikit, version 1 (Qiagen, Hilden, Germany) with the automated instrument QIAsimphony (Qiagen, Hilden, Germany) according to manufacturer’s instructions.

The genomic DNA of the 12 strains previously sequenced by Gona and colleagues^15^ were extracted using the DNeasy blood and tissue kit (Qiagen, Hilden, Germany) following the manufacturer’s instructions.

Details about strains hospital isolation are reported in Supplementary Table S3.

### High resolution melting primer design using EasyPrimer

The EasyPrimer tool was developed for the identification of the most suitable regions for primer design in HRM and, more in general, in pan-PCR experiments. Briefly, the tool starts from gene sequences in multi-fasta format. The sequences are considered as not aligned by default and they are aligned by Muscle software^22^ as the first step of the pipeline (see Supplementary Fig. S7 and Supplementary Note S1). The user can also decide to submit aligned sequences (in multi-fasta format) and skip the alignment step. EasyPrimer evaluates the amount of genetic variation for each position of the alignment and identifies the most reliable regions for primer design. EasyPrimer flags as good candidates for primer design two conserved regions flanking a highly variable one (taking into consideration, in advance, the optimal lengths of primers and amplicon). The user can decide either to evaluate the variability of the amplicon considering HRM-detectable SNPs only (the best option for HRM primer design) or all the SNPs (the best setting for pan-PCR experiments). A detailed description of the algorithm is reported in the Supplementary Note S1.

To develop an HRM-based protocol for *K. pneumoniae* typing, we focused on the seven MLST genes and on the hypervariable capsular gene *wzi*^23^. The HRM primer pairs for five out of the seven *K. pneumoniae* MLST genes were already available in literature^7^ (*infB, mdh, phoE, rpoB* and two pairs on *tonB*). For the remaining two MLST genes (*pgi* and *gapA*) and for the *wzi* capsular gene (two primer pairs) the primers were designed using EasyPrimer. For each gene, the sequences were downloaded from the BigsDB database (https://bigsdb.pasteur.fr,218 alleles for *pgi*, 183 for *gapA* and 563 for *wzi*), EasyPrimer was run and primer pairs were designed on the basis of its output.

### High-resolution melting assays

We performed HRM assays using the genomic DNA extracted from each of the 82 *K. pneumoniae* strains included in this work. On the strains of the background and outbreak collections we used each of the ten primer pairs mentioned above. On the validation collection strains we used *wzi-3* and *wzi-4* primer pairs only. HRM analyses were performed on the BioRad CFX Connect real-time PCR System (BioRad, Hercules, California). Each 10 µl reaction contained: 5 µl of 2x SsoAdvanced Universal SYBR® Green Supermix (BioRad, Hercules, California), 0.4 µl of each primer (0.4 µM) and 1 µl of template DNA (25–50 ng/µl). The thermal profile was as follows: 98 °C for 2 min, 40 cycles of [95 °C for 7 s, 61 °C for 7 s, and 72 °C for 15 s], 95 °C for 2 min, followed by HRM ramping from 70–95 °C with fluorescence data acquisition at 0.5 °C increments. Three technical replicates were performed for each strain and for each gene analysed. Negative controls were added in every run and for each gene.

### Comparison of the HRM primer pairs and schemes

We compared the discriminatory power of the ten HRM primer pairs on the 17 background collection strains. For each primer pair we calculated the average melting temperatures (aTms) of the three replicates for each strain and the relative strain distance matrix based on the obtained aTms. Thus, we compared the discriminatory power of the different primer pairs by comparing the relative distance matrix values using Wilcoxon test with Holm post-hoc correction.

Furthermore, we grouped the primer pairs in three schemes (MLST6, MLST8 and wzi) and we compared the relative strain distance matrices using Wilcoxon test with Holm post-hoc correction. The scheme compositions were as follows: the MLST6 scheme included the six primer pairs proposed by Andersson and colleagues^7^ for five MLST genes (with two primer pairs for *tonB*); the MLST8 included all the MLST6 primer pairs, the primers for *pgi* and *gapA* (newly designed in this work using the EasyPrimer tool); the wzi scheme included the two primer pairs for the *wzi* gene (newly designed in this work). For details see Table 1 and Fig. 2.

Then, we compared the discriminatory power of MLST8 and wzi schemes by subtracting the relative distance matrices (wzi – MLST8) and studying the obtained matrix with a heatmap.

All these analyses were performed using R (https://www.r-project.org/) and the R libraries Ape and Gplots.

### HRM-based outbreak reconstruction

From the aTms of the outbreak and background collections we calculated the distance matrices for MLST6, MLST8 and wzi primer schemes (for more details see above) and clustered the strains using the hierarchical clustering method implemented in the Hclust function in R.

### Repeatability of the *wzi* HRM protocol

To test the repeatability of the *wzi* HRM typing protocol we analysed the wzi scheme aTms for all the strains of the three collections (17 background strains, 11 outbreak and 54 validation, see above). This allowed to compare the *wzi*-3 and *wzi*-4 aTms of multiple strains for each of the most epidemiologically relevant clades^11,12^ (eight ST258 clade 1, 26 ST258 Clade 2, 19 ST307, 12 ST101, two ST11 and three ST15 strains) (see Table 2 and Supplementary Table S3).

We clustered the strains on the basis of wzi scheme aTms. Given the 0.5 °C sensitivity of the machine, we considered the strains with differences both in *wzi*-3 and *wzi*-4 aTMs < 0.5 as indistinguishable. Thus, we built a strain-to-strain network, in which the indistinguishable strains pairs were connected. Clusters were extracted from the network using the decompose igraph R function (https://www.r-project.org/). Lastly, the strain-to-strain network was converted to a dendrogram and merged to *wzi* aTms in a heatmap plot, using R (https://www.r-project.org/).

### WGS-based strain typing

We retrieved the genome assemblies of the 12 *K. pneumoniae* strains previously WGS-typed by Gaiarsa and colleagues^14^ from NCBI database and the reads files of the 12 strains by Gona and colleagues^15^ using fastq-dump tool (accession numbers are reported in Supplementary Table S3).

We performed *de novo* assembly on the reads obtained from the 58 strains sequenced in this work and on the reads of the 12 strains retrived from Gona and colleagues^15^ using SPAdes software^24^.

We retrieved the sequences of the *K. pneumoniae* MLST gene alleles and the relative scheme tables from the BigsDB database. Thus, we determined the MLST profiles using an in-house Blastn-based Perl script.

We retrieved the *wzi* allele sequences from BigsDB database and we annotated the *wzi* allele present in each of the 82 genome assemblies included in the study by Blastn search and manual curation of the results.

We annotated the K-type of the 82 strains using Kaptive^23^ on the genome assemblies.

### Core-SNP-based phylogenetic reconstruction on outbreak strains

We aligned the reads obtained from the 11 outbreak strains against the NTUH_K2044 reference genome (accession number NC_016845.1), and performed the SNPs calling following the GATK best practice procedure. We masked SNPs localized within repeated regions, identified using MUMmer^25^, or prophages, identified using PhiSpy^26^, and we called the core-SNPs among the strains using an in-house Python script. Thus, we subjected the core-SNPs alignment to phylogenetic analysis as follows: the best evolutionary model was assessed by ModelTest-NG and phylogenetic reconstruction was performed using the selected best model, with RAxML8 software^27^. We evaluated the core-SNPs distances among the strains using the R Ape library (https://www.r-project.org/).

## Supplementary information

Supplemetal material.

## Data Availability

We deposited all Illumina sequence data from the 58 strains in NCBI’s Short Read Archive under BioProject ID ERP119329 and all Illumina data were deposited under BioProject ID PRJEB36171.
