# Supplementary material for: EasyPrimer: user-friendly tool for pan-PCR/HRM primers design. Development of an HRM protocol on wzi gene for fast Klebsiella pneumoniae typing
Source: Sci Rep. 2020 Jan 28;10:1307. doi: 10.1038/s41598-020-57742-z (PMC6987216; doi:10.1038/s41598-020-57742-z)
Supplement: Supplementary file 1 — Supplemetal material. [file 41598_2020_57742_MOESM1_ESM.pdf]

Supplementary Information of:

EasyPrimer: user-friendly tool for pan-PCR/HRM primers design. Development of an HRM protocol on *wzi* gene for fast *Klebsiella pneumoniae* typing.

Matteo Perini[1], Aurora Piazza[1], Simona Panelli[1], Domenico Di Carlo[1], Marta Corbella[2], Floriana Gona[3], Francesca Vailati[4], Piero Marone[2], Daniela Maria Cirillo[3], Claudio Farina[4], Gianvincenzo Zuccotti[1,5], Francesco Comandatore*[,1]

[1]Department of Biomedical and Clinical Sciences “L. Sacco”, Università di Milano, Pediatric Clinical Research Center “Romeo and Enrica Invernizzi”, Milan, 20157, Italy

[2]S.C. Microbiologia e Virologia, Fondazione IRCCS Policlinico San Matteo, Pavia, 27100, Italy

[3]Emerging Bacterial Pathogens Unit, Division of Immunology, Transplantation and Infectious Diseases, IRCCS San Raffaele Scientific Institute, Milan, 20132, Italy

[4]Microbiology Institute, A.S.S.T. "Papa Giovanni XXIII", Bergamo, 24127, Italy

[5]Department of Pediatrics, V. Buzzi Childrens' Hospital, Università di Milano, Milan, Italy.

*francesco.comandatore@unimi.it

Supplementary Note S1. The EasyPrimer algorithm.

EasyPrimer is a Python-R pipeline to assist the primer design procedure. it was developed to identify the best regions to design primers in an alignment. Indeed, the tool finds the best “primer-amplicon-primer” gene region, where two highly conserved gene regions (suggested for primer design) flank a highly variable region (the “amplicon region”).

The algorithm scans the alignment to find - at least - one highly variable region (amplicon region) flanked by two conserved regions (primer regions). For the computation of the highly variable region, the user can decide to consider all the Single Nucleotide Polymorphism (SNPs) or only the HRM-detectable ones (HRM-SNP): a HRM-SNP is a SNP that confers a change in CG content (i.e., any A↔G or T↔G or A↔C or T↔G counts as a HRM-SNP). EasyPrimer will identify the most HRM discriminatory region in the alignment when HRM SNPs are used to score the variability of the amplicon region. Conversely, considering all the SNPs the tool will identify the most variable region in general. Additionally, the user provides length ranges both for primers and amplicon, the tool suggests regions for primer design with a reasonable spacing, meaning that they always amplify a region of optimal length.

The main EasyPrimer output is a graph (plotted in a PDF file) in which the alignment consensus sequence is reported on the x-axes and the information about the positions of the best primer/amplicon regions are represented with blue and red curves.

The algorithm:

The inputs of the tool are:

i) A Fasta sequence of the gene to be analysed;

ii) A minimum and a maximum nucleotide length allowed for the primers and the amplicon;

iii) A percentile threshold of variability for primers selection (see below);

iv) A percentile threshold of variability for amplicon selection (see below);

v) A consensus threshold value (see below);

The steps are the following:

1. Aligning the gene sequences using the MUSCLE software (MUltiple Sequence Comparison by Log- Expectation)[1]
2. Computing, for each position of the alignment, the frequencies of A, T, C, G and gaps
3. Computing for each position the information of the entropy using Shannon index on the basis of A, T, C, G and gaps frequencies as follows:

$$H' = [f_A \times \log(f_A)] + [f_T \times \log(f_T)] + [f_C \times \log(f_C)] + [f_G \times \log(f_G)] + [f_{gaps} \times \log(f_{gaps})]$$

4. Estimating the frequency of mutation among strains. If the user decided to use all the possible SNPs to evaluate the amplicon variability the score is calculated as follows:

$$f_{SNP} = \frac{(f_A \times f_G) + (f_T \times f_G) + (f_A \times f_C) + (f_T \times f_C) + (f_A \times f_T) + (f_G \times f_C)}{amplicon\ length}$$

Otherwise, if the user decided to work with HRM-SNPs only the formula becomes:

$$f_{HRM} = \frac{(f_A \times f_G) + (f_T \times f_G) + (f_A \times f_C) + (f_T \times f_C)}{amplicon\ length}$$

5. For each possible combination of primers and amplicon lengths a window composed by primer-amplicon-primer sections is generated and shifted on the sequence alignment. For each window position, the average H' is calculated for the primer regions and the average f_{HRM} or f_{SNP} is calculated for the amplicon region
6. Selection of the windows with total H' below the primer percentile threshold in the primer region and f_{HRM} or f_{SNP} above the amplicon percentile threshold in the amplicon region.
7. The consensus of the alignment is obtained
8. The results are plotted on a graph in which the consensus sequence is reported on the x-axes and blue/red lines are obtained as follows: for each alignment position, the frequency of selected windows for which the position is included in the primer section is represented in blue, while the frequency for which the position in included in the amplicon section is represented in red

Notes on the consensus notation:

The consensus sequence is written the IUPAC notation for DNA both for the bases and the degenerated residues. some symbols and notation were added to give more information:

- Red residues are added to indicate in the consensus sequence that gaps in the alignment in that position are greater than consensus threshold (more than 5% in default) and less than 1 - consensus threshold (less than 95% in default).

- Asterisks (*) indicate a gap present more than 1 - consensus threshold (more than 95% in defaults), this indicate an insertion in one or few variances in the gene. The user will probably want to consider that position as ‘not existing’ during the primer design process

The algorithm is summarized in the flow diagram in Figure S7.

Reference:

1. Edgar, R. C. MUSCLE: multiple sequence alignment with high accuracy and high throughput. *Nucleic Acids Res*. **32**, 1792–1797 (2004).

Table S1. List of all the melting temperatures obtained during the HRM analyses for all the strains and for all the primer sets.

Strain name	MLST profile	Gene name	Primer pair name	Melting T 1 (°C)	Melting T 2 (°C)	Melting T 3 (°C)	Average melting T (°C)
15PV	ST976	*infB*	*infB-1*	84	84	84	84
15PV	ST976	*mdh*	*mdh-1*	80.5	80.5	80.5	80.5
15PV	ST976	*phoE*	*phoE-1*	80	80	80	80
15PV	ST976	*rpoB*	*rpoB-1*	83.5	83.5	83	83.3
15PV	ST976	*tonB*	*tonB-1*	86.5	86.5	86	86.3
15PV	ST976	*tonB*	*tonB-2*	89	89	89	89
15PV	ST976	*gapA*	*gapA-1*	84.5	84.5	84.5	84.5
15PV	ST976	*pgi*	*pgi-1*	86	86	86	86
15PV	ST976	*wzi*	*wzi-3*	85	85	85	85
15PV	ST976	*wzi*	*wzi-4*	83.5	83.5	83.5	83.5
17PV	ST307	*pgi*	*pgi-1*	86	85.5	85.5	85.7
17PV	ST307	*wzi*	*wzi-3*	84.5	84	84	84.2
17PV	ST307	*wzi*	*wzi-4*	82.5	82.5	82.5	82.5
17PV	ST307	*infB*	*infB-1*	84	83.5	83.5	83.7
17PV	ST307	*mdh*	*mdh-1*	80	80	80	80
17PV	ST307	*phoE*	*phoE-1*	81	81	80.5	80.8
17PV	ST307	*rpoB*	*rpoB-1*	83.5	83.5	83.5	83.5
17PV	ST307	*tonB*	*tonB-1*	86.5	86	86	86.2
17PV	ST307	*tonB*	*tonB-2*	89.5	89.5	89.5	89.5
17PV	ST307	*gapA*	*gapA-1*	85.5	85	85	85.2
21PV	ST307	*infB*	*infB-1*	84.5	84.5	84	84.3
21PV	ST307	*mdh*	*mdh-1*	80.5	80.5	80.5	80.5
21PV	ST307	*phoE*	*phoE-1*	81	81	81	81
21PV	ST307	*rpoB*	*rpoB-1*	83.5	83.5	83.5	83.5
21PV	ST307	*tonB*	*tonB-1*	86.5	86.5	86.5	86.5
21PV	ST307	*tonB*	*tonB-2*	89	89	89	89
21PV	ST307	*gapA*	*gapA-1*	85	85	85	85
21PV	ST307	*pgi*	*pgi-1*	86	86	86	86
21PV	ST307	*wzi*	*wzi-3*	85	85	85	85
21PV	ST307	*wzi*	*wzi-4*	83.5	83.5	83.5	83.5
49BG	ST147	*infB*	*infB-1*	83.5	83.5	83.5	83.5
49BG	ST147	*mdh*	*mdh-1*	79.5	80	79.5	79.7

Strain name	MLST profile	Gene name	Primer pair name	Melting T 1 (°C)	Melting T 2 (°C)	Melting T 3 (°C)	Average melting T (°C)
49BG	ST147	*phoE*	*phoE-1*	80.5	80.5	80.5	80.5
49BG	ST147	*rpoB*	*rpoB-1*	83.5	83.5	84	83.7
49BG	ST147	*tonB*	*tonB-1*	89	89	89	89
49BG	ST147	*tonB*	*tonB-2*	86.5	86.5	86.5	86.5
49BG	ST147	*gapA*	*gapA-1*	85.5	85	85	85.2
49BG	ST147	*pgi*	*pgi-1*	86	86	86	86
49BG	ST147	*wzi*	*wzi-3*	85	85	85	85
49BG	ST147	*wzi*	*wzi-4*	84.5	84.5	84.5	84.5
51BG	ST1626	*infB*	*infB-1*	83.5	83.5	83	83.3
51BG	ST1626	*mdh*	*mdh-1*	80	80	80	80
51BG	ST1626	*phoE*	*phoE-1*	80.5	80.5	80.5	80.5
51BG	ST1626	*rpoB*	*rpoB-1*	83.5	83.5	83.5	83.5
51BG	ST1626	*tonB*	*tonB-1*	86	86	86	86
51BG	ST1626	*tonB*	*tonB-2*	89	89	89	89
51BG	ST1626	*gapA*	*gapA-1*	85	85	85	85
51BG	ST1626	*pgi*	*pgi-1*	86	86	86	86
51BG	ST1626	*wzi*	*wzi-3*	85	85	85	85
51BG	ST1626	*wzi*	*wzi-4*	84	84	84	84
52BG	ST10	*infB*	*infB-1*	83.5	83.5	83.5	83.5
52BG	ST10	*mdh*	*mdh-1*	78.5	79	79	78.8
52BG	ST10	*phoE*	*phoE-1*	80	80.5	80	80.2
52BG	ST10	*rpoB*	*rpoB-1*	83	83	83.5	83.2
52BG	ST10	*tonB*	*tonB-1*	86	86	86	86
52BG	ST10	*tonB*	*tonB-2*	88.5	89	89	88.8
52BG	ST10	*gapA*	*gapA-1*	84.5	85	84.5	84.7
52BG	ST10	*pgi*	*pgi-1*	86	86	86	86
52BG	ST10	*wzi*	*wzi-3*	85	85	85	85
52BG	ST10	*wzi*	*wzi-4*	84.5	84.5	84.5	84.5
53BG	ST321	*infB*	*infB-1*	84	84.5	84	84.2
53BG	ST321	*mdh*	*mdh-1*	80	80	80	80
53BG	ST321	*phoE*	*phoE-1*	81	81	81	81
53BG	ST321	*rpoB*	*rpoB-1*	83	83	83	83
53BG	ST321	*tonB*	*tonB-1*	86.5	86.5	86.5	86.5
53BG	ST321	*tonB*	*tonB-2*	89	89	89	89
53BG	ST321	*gapA*	*gapA-1*	85	85	85	85

Strain name	MLST profile	Gene name	Primer pair name	Melting T 1 (°C)	Melting T 2 (°C)	Melting T 3 (°C)	Average melting T (°C)
53BG	ST321	*pgi*	*pgi-1*	86	86	86	86
53BG	ST321	*wzi*	*wzi-3*	85	85	85	85
53BG	ST321	*wzi*	*wzi-4*	84	84	84	84
55BG	ST466	*infB*	*infB-1*	83.5	83.5	83	83.3
55BG	ST466	*mdh*	*mdh-1*	80.5	80.5	80.5	80.5
55BG	ST466	*phoE*	*phoE-1*	80.5	80.5	80.5	80.5
55BG	ST466	*rpoB*	*rpoB-1*	83.5	83.5	83.5	83.5
55BG	ST466	*tonB*	*tonB-1*	86.5	86.5	86.5	86.5
55BG	ST466	*tonB*	*tonB-2*	89	89	89	89
55BG	ST466	*gapA*	*gapA-1*	85	85	85	85
55BG	ST466	*pgi*	*pgi-1*	86	86	86	86
55BG	ST466	*wzi*	*wzi-3*	83.5	83.5	83.5	83.5
55BG	ST466	*wzi*	*wzi-4*	84.5	84.5	84.5	84.5
63BG	ST1627	*infB*	*infB-1*	83.5	83.5	83	83.3
63BG	ST1627	*mdh*	*mdh-1*	80.5	80.5	80.5	80.5
63BG	ST1627	*phoE*	*phoE-1*	80.5	80.5	80.5	80.5
63BG	ST1627	*rpoB*	*rpoB-1*	83.5	83.5	83.5	83.5
63BG	ST1627	*tonB*	*tonB-1*	86.5	86.5	86.5	86.5
63BG	ST1627	*tonB*	*tonB-2*	88.5	89.5	89.5	89.2
63BG	ST1627	*gapA*	*gapA-1*	85	85	85	85
63BG	ST1627	*pgi*	*pgi-1*	86	86	86.5	86.2
63BG	ST1627	*wzi*	*wzi-3*	84	84	84	84
63BG	ST1627	*wzi*	*wzi-4*	84.5	84.5	84.5	84.5
BG-Kpn-20-18	ST512	*wzi*	*wzi-3*	84	84	84	84
BG-Kpn-20-18	ST512	*wzi*	*wzi-4*	83.5	83.5	83.5	83.5
BG-Kpn-20-18	ST512	*pgi*	*pgi-1*	86	86	86	86
BG-Kpn-20-18	ST512	*infB*	*infB-1*	83.5	83.5	83.5	83.5
BG-Kpn-20-18	ST512	*mdh*	*mdh-1*	80.5	80.5	80.5	80.5
BG-Kpn-20-18	ST512	*phoE*	*phoE-1*	80.5	80.5	80.5	80.5
BG-Kpn-20-18	ST512	*rpoB*	*rpoB-1*	83.5	83.5	83	83.3
BG-Kpn-20-18	ST512	*tonB*	*tonB-1*	86.5	86.5	86.5	86.5
BG-Kpn-20-18	ST512	*tonB*	*tonB-2*	88.5	88.5	88.5	88.5
BG-Kpn-20-18	ST512	*gapA*	*gapA-1*	85	84.5	84.5	84.7
BG-Kpn-21-18	ST512	*pgi*	*pgi-1*	86.5	86	86	86.2
BG-Kpn-21-18	ST512	*wzi*	*wzi-3*	84	84	84	84

Strain name	MLST profile	Gene name	Primer pair name	Melting T 1 (°C)	Melting T 2 (°C)	Melting T 3 (°C)	Average melting T (°C)
BG-Kpn-21-18	ST512	*wzi*	*wzi-4*	83.5	83.5	83.5	83.5
BG-Kpn-21-18	ST512	*infB*	*infB-1*	83.5	83.5	83.5	83.5
BG-Kpn-21-18	ST512	*mdh*	*mdh-1*	80.5	80.5	80.5	80.5
BG-Kpn-21-18	ST512	*phoE*	*phoE-1*	80.5	80.5	80.5	80.5
BG-Kpn-21-18	ST512	*rpoB*	*rpoB-1*	83.5	83.5	83.5	83.5
BG-Kpn-21-18	ST512	*tonB*	*tonB-1*	86.5	86.5	86.5	86.5
BG-Kpn-21-18	ST512	*tonB*	*tonB-2*	88.5	88.5	88.5	88.5
BG-Kpn-21-18	ST512	*gapA*	*gapA-1*	85	85	85	85
BG-Kpn-22-18	ST307	*pgi*	*pgi-1*	86	86	86	86
BG-Kpn-22-18	ST307	*wzi*	*wzi-3*	84.5	84.5	84.5	84.5
BG-Kpn-22-18	ST307	*wzi*	*wzi-4*	82.5	82.5	82.5	82.5
BG-Kpn-22-18	ST307	*infB*	*infB-1*	84	84	84	84
BG-Kpn-22-18	ST307	*mdh*	*mdh-1*	79.5	79.5	79.5	79.5
BG-Kpn-22-18	ST307	*phoE*	*phoE-1*	80.5	80.5	80.5	80.5
BG-Kpn-22-18	ST307	*rpoB*	*rpoB-1*	83.5	83.5	83	83.3
BG-Kpn-22-18	ST307	*tonB*	*tonB-1*	86.5	86.5	86.5	86.5
BG-Kpn-22-18	ST307	*tonB*	*tonB-2*	89	89	89	89
BG-Kpn-22-18	ST307	*gapA*	*gapA-1*	85	85	85	85
BG-Kpn-23-18	ST512	*pgi*	*pgi-1*	86	86	86.5	86.2
BG-Kpn-23-18	ST512	*wzi*	*wzi-3*	84	84	84	84
BG-Kpn-23-18	ST512	*wzi*	*wzi-4*	83.5	83.5	83.5	83.5
BG-Kpn-23-18	ST512	*infB*	*infB-1*	83	83	83	83
BG-Kpn-23-18	ST512	*mdh*	*mdh-1*	80.5	80.5	80.5	80.5
BG-Kpn-23-18	ST512	*phoE*	*phoE-1*	80.5	80.5	80.5	80.5
BG-Kpn-23-18	ST512	*rpoB*	*rpoB-1*	83.5	83.5	83.5	83.5
BG-Kpn-23-18	ST512	*tonB*	*tonB-1*	86.5	86.5	86.5	86.5
BG-Kpn-23-18	ST512	*tonB*	*tonB-2*	88.5	88.5	88.5	88.5
BG-Kpn-23-18	ST512	*gapA*	*gapA-1*	84.5	84.5	84.5	84.5
BG-Kpn-24-18	ST512	*pgi*	*pgi-1*	86.5	86	86	86.2
BG-Kpn-24-18	ST512	*wzi*	*wzi-3*	84	84	84	84
BG-Kpn-24-18	ST512	*wzi*	*wzi-4*	83.5	83.5	83.5	83.5
BG-Kpn-24-18	ST512	*infB*	*infB-1*	83.5	83.5	83.5	83.5
BG-Kpn-24-18	ST512	*mdh*	*mdh-1*	80.5	80.5	80.5	80.5
BG-Kpn-24-18	ST512	*phoE*	*phoE-1*	81	81	81	81
BG-Kpn-24-18	ST512	*rpoB*	*rpoB-1*	83.5	83.5	83.5	83.5

Strain name	MLST profile	Gene name	Primer pair name	Melting T 1 (°C)	Melting T 2 (°C)	Melting T 3 (°C)	Average melting T (°C)
BG-Kpn-24-18	ST512	*tonB*	*tonB-1*	86.5	86.5	86.5	86.5
BG-Kpn-24-18	ST512	*tonB*	*tonB-2*	89	89	89	89
BG-Kpn-24-18	ST512	*gapA*	*gapA-1*	84.5	84.5	84.5	84.5
BG-Kpn-25-18	ST512	*pgi*	*pgi-1*	86	86	86	86
BG-Kpn-25-18	ST512	*wzi*	*wzi-3*	84	84	84	84
BG-Kpn-25-18	ST512	*wzi*	*wzi-4*	83.5	83.5	83.5	83.5
BG-Kpn-25-18	ST512	*infB*	*infB-1*	83.5	83.5	83.5	83.5
BG-Kpn-25-18	ST512	*mdh*	*mdh-1*	80.5	80.5	80.5	80.5
BG-Kpn-25-18	ST512	*phoE*	*phoE-1*	81	81	81	81
BG-Kpn-25-18	ST512	*rpoB*	*rpoB-1*	83.5	83.5	83.5	83.5
BG-Kpn-25-18	ST512	*tonB*	*tonB-1*	86.5	86.5	86.5	86.5
BG-Kpn-25-18	ST512	*tonB*	*tonB-2*	88.5	88.5	88.5	88.5
BG-Kpn-25-18	ST512	*gapA*	*gapA-1*	84.5	84.5	84.5	84.5
BG-Kpn-26-18	ST512	*pgi*	*pgi-1*	86	86	86	86
BG-Kpn-26-18	ST512	*wzi*	*wzi-3*	84	84	84	84
BG-Kpn-26-18	ST512	*wzi*	*wzi-4*	83.5	83.5	83.5	83.5
BG-Kpn-26-18	ST512	*infB*	*infB-1*	83.5	83.5	83.5	83.5
BG-Kpn-26-18	ST512	*mdh*	*mdh-1*	80.5	80.5	80.5	80.5
BG-Kpn-26-18	ST512	*phoE*	*phoE-1*	81	81	80.5	80.8
BG-Kpn-26-18	ST512	*rpoB*	*rpoB-1*	83.5	83.5	83.5	83.5
BG-Kpn-26-18	ST512	*tonB*	*tonB-1*	86.5	86.5	86.5	86.5
BG-Kpn-26-18	ST512	*tonB*	*tonB-2*	88.5	88.5	88.5	88.5
BG-Kpn-26-18	ST512	*gapA*	*gapA-1*	84.5	84.5	84.5	84.5
BG-Kpn-27-18	ST512	*pgi*	*pgi-1*	86	86	86	86
BG-Kpn-27-18	ST512	*wzi*	*wzi-3*	84	84	84	84
BG-Kpn-27-18	ST512	*wzi*	*wzi-4*	83.5	83.5	83.5	83.5
BG-Kpn-27-18	ST512	*infB*	*infB-1*	83.5	83.5	83.5	83.5
BG-Kpn-27-18	ST512	*mdh*	*mdh-1*	80.5	80.5	80.5	80.5
BG-Kpn-27-18	ST512	*phoE*	*phoE-1*	81	81	81	81
BG-Kpn-27-18	ST512	*rpoB*	*rpoB-1*	83.5	83.5	83.5	83.5
BG-Kpn-27-18	ST512	*tonB*	*tonB-1*	86.5	86.5	86.5	86.5
BG-Kpn-27-18	ST512	*tonB*	*tonB-2*	88.5	88.5	89	88.7
BG-Kpn-27-18	ST512	*gapA*	*gapA-1*	84.5	84.5	84.5	84.5
BG-Kpn-28-18	ST512	*pgi*	*pgi-1*	86	86	86	86
BG-Kpn-28-18	ST512	*wzi*	*wzi-3*	84	84	84	84

**Strain name**	**MLST profile**	**Gene name**	**Primer pair name**	**Melting T 1 (°C)**	**Melting T 2 (°C)**	**Melting T 3 (°C)**	**Average melting T (°C)**
BG-Kpn-28-18	ST512	*wzi*	*wzi-4*	83.5	83.5	83.5	83.5
BG-Kpn-28-18	ST512	*infB*	*infB-1*	83.5	83	83.5	83.3
BG-Kpn-28-18	ST512	*mdh*	*mdh-1*	80.5	80.5	80.5	80.5
BG-Kpn-28-18	ST512	*phoE*	*phoE-1*	81	81	81	81
BG-Kpn-28-18	ST512	*rpoB*	*rpoB-1*	83.5	83.5	83.5	83.5
BG-Kpn-28-18	ST512	*tonB*	*tonB-1*	86.5	86.5	86.5	86.5
BG-Kpn-28-18	ST512	*tonB*	*tonB-2*	89	89	89	89
BG-Kpn-28-18	ST512	*gapA*	*gapA-1*	84.5	84.5	84.5	84.5
BG-Kpn-29-18	ST512	*pgi*	*pgi-1*	86	86	86	86
BG-Kpn-29-18	ST512	*wzi*	*wzi-3*	84	84	84	84
BG-Kpn-29-18	ST512	*wzi*	*wzi-4*	83.5	83.5	83.5	83.5
BG-Kpn-29-18	ST512	*infB*	*infB-1*	83.5	83.5	83.5	83.5
BG-Kpn-29-18	ST512	*mdh*	*mdh-1*	80.5	80.5	80.5	80.5
BG-Kpn-29-18	ST512	*phoE*	*phoE-1*	81	81	81	81
BG-Kpn-29-18	ST512	*rpoB*	*rpoB-1*	83.5	83.5	83.5	83.5
BG-Kpn-29-18	ST512	*tonB*	*tonB-1*	86.5	86.5	86.5	86.5
BG-Kpn-29-18	ST512	*gapA*	*gapA-1*	84.5	84.5	84.5	84.5
BG-Kpn-29-18	ST512	*tonB*	*tonB-2*	89	89	89	89
BG-Kpn-30-18	ST512	*pgi*	*pgi-1*	86	86	86	86
BG-Kpn-30-18	ST512	*wzi*	*wzi-3*	84	84	84	84
BG-Kpn-30-18	ST512	*wzi*	*wzi-4*	83.5	83.5	83.5	83.5
BG-Kpn-30-18	ST512	*infB*	*infB-1*	83.5	83.5	83.5	83.5
BG-Kpn-30-18	ST512	*mdh*	*mdh-1*	80.5	80.5	80.5	80.5
BG-Kpn-30-18	ST512	*phoE*	*phoE-1*	81	81	81	81
BG-Kpn-30-18	ST512	*rpoB*	*rpoB-1*	83.5	83.5	83.5	83.5
BG-Kpn-30-18	ST512	*tonB*	*tonB-1*	86.5	86.5	86.5	86.5
BG-Kpn-30-18	ST512	*tonB*	*tonB-2*	89	89	89	89
BG-Kpn-30-18	ST512	*gapA*	*gapA-1*	84.5	84.5	84.5	84.5
KP123	ST512	*infB*	*infB-1*	83.5	83.5	83	83.3
KP123	ST512	*mdh*	*mdh-1*	80.5	80.5	80.5	80.5
KP123	ST512	*phoE*	*phoE-1*	81	81	80.5	80.8
KP123	ST512	*rpoB*	*rpoB-1*	83.5	83.5	83.5	83.5
KP123	ST512	*tonB*	*tonB-1*	86.5	86.5	86.5	86.5
KP123	ST512	*tonB*	*tonB-2*	88.5	88.5	88.5	88.5
KP123	ST512	*gapA*	*gapA-1*	84.5	84.5	84.5	84.5

Strain name	MLST profile	Gene name	Primer pair name	Melting T 1 (°C)	Melting T 2 (°C)	Melting T 3 (°C)	Average melting T (°C)
KP123	ST512	*pgi*	*pgi-1*	86	86	86	86
KP123	ST512	*wzi*	*wzi-3*	84	84	84	84
KP123	ST512	*wzi*	*wzi-4*	83.5	83.5	83.5	83.5
KP248	ST423	*infB*	*infB-1*	83.5	83.5	83.5	83.5
KP248	ST423	*mdh*	*mdh-1*	80.5	80.5	80.5	80.5
KP248	ST423	*phoE*	*phoE-1*	81	81	80.5	80.8
KP248	ST423	*rpoB*	*rpoB-1*	83.5	83.5	83.5	83.5
KP248	ST423	*tonB*	*tonB-1*	86.5	86.5	86.5	86.5
KP248	ST423	*tonB*	*tonB-2*	89	89	89	89
KP248	ST423	*gapA*	*gapA-1*	84.5	84.5	84.5	84.5
KP248	ST423	*pgi*	*pgi-1*	86	86	86	86
KP248	ST423	*wzi*	*wzi-3*	84	84	83.5	83.8
KP248	ST423	*wzi*	*wzi-4*	83.5	83.5	83.5	83.5
KP261	ST258	*infB*	*infB-1*	83.5	83.5	83.5	83.5
KP261	ST258	*mdh*	*mdh-1*	80.5	80.5	80.5	80.5
KP261	ST258	*phoE*	*phoE-1*	80.5	80.5	80.5	80.5
KP261	ST258	*rpoB*	*rpoB-1*	83.5	83.5	83.5	83.5
KP261	ST258	*tonB*	*tonB-1*	86.5	86.5	86.5	86.5
KP261	ST258	*tonB*	*tonB-2*	88.5	88.5	88.5	88.5
KP261	ST258	*gapA*	*gapA-1*	85	85	85	85
KP261	ST258	*pgi*	*pgi-1*	86	86	86	86
KP261	ST258	*wzi*	*wzi-3*	83.5	83.5	83.5	83.5
KP261	ST258	*wzi*	*wzi-4*	84.5	84.5	84.5	84.5
KP359	ST149	*infB*	*infB-1*	84	84	84	84
KP359	ST149	*mdh*	*mdh-1*	80	80	80	80
KP359	ST149	*phoE*	*phoE-1*	81	81	80.5	80.8
KP359	ST149	*rpoB*	*rpoB-1*	84	84	84	84
KP359	ST149	*tonB*	*tonB-1*	86.5	86.5	86.5	86.5
KP359	ST149	*tonB*	*tonB-2*	89	89	89	89
KP359	ST149	*gapA*	*gapA-1*	85	85	85	85
KP359	ST149	*pgi*	*pgi-1*	86	86	86	86
KP359	ST149	*wzi*	*wzi-3*	84	84	84	84
KP359	ST149	*wzi*	*wzi-4*	84	84	84	84
KP366	ST11	*infB*	*infB-1*	83.5	83.5	83	83.3
KP366	ST11	*mdh*	*mdh-1*	80.5	80.5	80.5	80.5

**Strain name**	**MLST profile**	**Gene name**	**Primer pair name**	**Melting T 1 (°C)**	**Melting T 2 (°C)**	**Melting T 3 (°C)**	**Average melting T (°C)**
KP366	ST11	*phoE*	*phoE-1*	81	80.5	80.5	80.7
KP366	ST11	*rpoB*	*rpoB-1*	83.5	83.5	83.5	83.5
KP366	ST11	*tonB*	*tonB-1*	87	87	87	87
KP366	ST11	*tonB*	*tonB-2*	88.5	88.5	88.5	88.5
KP366	ST11	*pgi*	*pgi-1*	86	86	86	86
KP366	ST11	*gapA*	*gapA-1*	85	85	85	85
KP366	ST11	*wzi*	*wzi-3*	85	85	85	85
KP366	ST11	*wzi*	*wzi-4*	84	84	84	84
KP469	ST395	*infB*	*infB-1*	84	84	84	84
KP469	ST395	*mdh*	*mdh-1*	80	80	80	80
KP469	ST395	*phoE*	*phoE-1*	81	80.5	80.5	80.7
KP469	ST395	*rpoB*	*rpoB-1*	83.5	83.5	83.5	83.5
KP469	ST395	*tonB*	*tonB-1*	87	87	87	87
KP469	ST395	*tonB*	*tonB-2*	89	89	89	89
KP469	ST395	*gapA*	*gapA-1*	85	85	85	85
KP469	ST395	*pgi*	*pgi-1*	85.5	85.5	85.5	85.5
KP469	ST395	*wzi*	*wzi-3*	85	85	85	85
KP469	ST395	*wzi*	*wzi-4*	83.5	83.5	83.5	83.5
KP473	ST101	*infB*	*infB-1*	84	84	84	84
KP473	ST101	*mdh*	*mdh-1*	80.5	80.5	80.5	80.5
KP473	ST101	*phoE*	*phoE-1*	80.5	80.5	80.5	80.5
KP473	ST101	*rpoB*	*rpoB-1*	83.5	83.5	83.5	83.5
KP473	ST101	*tonB*	*tonB-1*	86.5	86.5	86.5	86.5
KP473	ST101	*tonB*	*tonB-2*	89	89	88.5	88.8
KP473	ST101	*pgi*	*pgi-1*	86	86	86	86
KP473	ST101	*gapA*	*gapA-1*	85	85	85	85
KP473	ST101	*wzi*	*wzi-3*	85	85	85	85
KP473	ST101	*wzi*	*wzi-4*	84	84	83.5	83.8
KP485	ST15	*infB*	*infB-1*	84	84	84	84
KP485	ST15	*mdh*	*mdh-1*	80.5	80.5	80.5	80.5
KP485	ST15	*phoE*	*phoE-1*	81	80.5	80.5	80.7
KP485	ST15	*rpoB*	*rpoB-1*	83.5	83.5	83.5	83.5
KP485	ST15	*tonB*	*tonB-1*	86.5	86.5	86.5	86.5
KP485	ST15	*tonB*	*tonB-2*	89	89	89	89
KP485	ST15	*gapA*	*gapA-1*	85	85	85	85

Strain name	MLST profile	Gene name	Primer pair name	Melting T 1 (°C)	Melting T 2 (°C)	Melting T 3 (°C)	Average melting T (°C)
KP485	ST15	*pgi*	*pgi-1*	86	86	86	86
KP485	ST15	*wzi*	*wzi-3*	82.5	82.5	82	82.3
KP485	ST15	*wzi*	*wzi-4*	84	84	84	84
54BG	ST258	*wzi*	*wzi-3*	84	84	84	84
54BG	ST258	*wzi*	*wzi-4*	85	84.5	84.5	84.7
56BG	ST258	*wzi*	*wzi-4*	84.5	84.5	84.5	84.5
56BG	ST258	*wzi*	*wzi-3*	84	84	84	84
57BG	ST258	*wzi*	*wzi-3*	84	83.5	83.5	83.7
57BG	ST258	*wzi*	*wzi-4*	84.5	84.5	84.5	84.5
BG-Kpn-101-19	ST15	*wzi*	*wzi-3*	85.5	85	85	85.2
BG-Kpn-101-19	ST15	*wzi*	*wzi-4*	84	84	83.5	83.8
BG-Kpn-103-19	ST15	*wzi*	*wzi-3*	85	85	85	85
BG-Kpn-103-19	ST15	*wzi*	*wzi-4*	84	84	83.5	83.8
KP1-19	ST101	*wzi*	*wzi-3*	85	85	85	85
KP1-19	ST101	*wzi*	*wzi-4*	83.5	83.5	83.5	83.5
KP108-18-2	ST512	*wzi*	*wzi-3*	83.5	83.5	83.5	83.5
KP108-18-2	ST512	*wzi*	*wzi-4*	83.5	83.5	83	83.3
KP11-19	ST512	*wzi*	*wzi-3*	83.5	83.5	83.5	83.5
KP11-19	ST512	*wzi*	*wzi-4*	83.5	83.5	83	83.3
KP12-19	ST512	*wzi*	*wzi-4*	83.5	83.5	83.5	83.5
KP12-19	ST512	*wzi*	*wzi-3*	83.5	83.5	83.5	83.5
KP12-19-2	ST512	*wzi*	*wzi-3*	83.5	83.5	83.5	83.5
KP12-19-2	ST512	*wzi*	*wzi-4*	83.5	83.5	83.5	83.5
KP13-19	ST307	*wzi*	*wzi-4*	82.5	82.5	82.5	82.5
KP13-19	ST307	*wzi*	*wzi-3*	84	84	84	84
KP14-19	ST512	*wzi*	*wzi-4*	83.5	83.5	83.5	83.5
KP14-19	ST512	*wzi*	*wzi-3*	83.5	83.5	83.5	83.5
KP15-19	ST307	*wzi*	*wzi-3*	84.5	84.5	84.5	84.5
KP35-19	ST307	*wzi*	*wzi-3*	84.5	84.5	84.5	84.5
KP17-19	ST101	*wzi*	*wzi-4*	83.5	83.5	83.5	83.5
KP17-19	ST101	*wzi*	*wzi-3*	85	85	84.5	84.8
KP18-19	ST101	*wzi*	*wzi-4*	83.5	83.5	83.5	83.5
KP18-19	ST101	*wzi*	*wzi-3*	85	85	84.5	84.8
KP19-19	ST512	*wzi*	*wzi-4*	83.5	83.5	83.5	83.5
KP19-19	ST512	*wzi*	*wzi-3*	83.5	83.5	83.5	83.5

Strain name	MLST profile	Gene name	Primer pair name	Melting T 1 (°C)	Melting T 2 (°C)	Melting T 3 (°C)	Average melting T (°C)
KP2-19	ST307	*wzi*	*wzi-4*	82.5	82.5	82.5	82.5
KP2-19	ST307	*wzi*	*wzi-3*	84	84	84	84
KP20-19	ST258	*wzi*	*wzi-3*	83.5	83.5	83.5	83.5
KP20-19	ST258	*wzi*	*wzi-4*	84.5	84.5	84	84.3
KP21-19	ST258	*wzi*	*wzi-3*	83.5	83.5	83.5	83.5
KP21-19	ST258	*wzi*	*wzi-4*	84.5	84.5	84.5	84.5
KP232	ST258	*wzi*	*wzi-3*	84	84	83.5	83.8
KP232	ST258	*wzi*	*wzi-4*	83.5	83.5	83.5	83.5
KP24-19	ST512	*wzi*	*wzi-3*	83.5	83.5	83.5	83.5
KP24-19	ST512	*wzi*	*wzi-4*	83.5	83.5	83.5	83.5
KP246	ST258	*wzi*	*wzi-4*	84.5	84.5	84.5	84.5
KP246	ST258	*wzi*	*wzi-3*	84	84	84	84
KP25-19	ST307	*wzi*	*wzi-3*	84.5	84.5	84	84.3
KP25-19	ST307	*wzi*	*wzi-4*	82.5	82.5	82.5	82.5
KP252	ST258	*wzi*	*wzi-3*	84	84	83.5	83.8
KP252	ST258	*wzi*	*wzi-4*	83.5	83.5	83.5	83.5
KP26-19	ST307	*wzi*	*wzi-4*	83	82.5	82.5	82.7
KP26-19	ST307	*wzi*	*wzi-3*	84.5	84.5	84.5	84.5
KP27-19	ST307	*wzi*	*wzi-4*	83	82.5	82.5	82.7
KP27-19	ST307	*wzi*	*wzi-3*	84.5	84.5	84.5	84.5
KP29-19	ST512	*wzi*	*wzi-4*	83.5	83.5	83	83.3
KP29-19	ST512	*wzi*	*wzi-3*	83.5	83.5	83.5	83.5
KP3-19	ST307	*wzi*	*wzi-4*	82.5	82.5	82.5	82.5
KP3-19	ST307	*wzi*	*wzi-3*	84.5	84	84	84.2
KP34-19	ST101	*wzi*	*wzi-4*	83.5	83.5	83.5	83.5
KP34-19	ST101	*wzi*	*wzi-3*	85	85	85	85
KP37-19	ST307	*wzi*	*wzi-3*	84.5	84.5	84.5	84.5
KP4-19	ST101	*wzi*	*wzi-3*	85	85	85	85
KP36-19	ST101	*wzi*	*wzi-4*	83.5	83.5	83.5	83.5
KP36-19	ST101	*wzi*	*wzi-3*	85	85	85	85
KP65-18-3	ST101	*wzi*	*wzi-3*	85	85	84.5	84.8
KP8-19	ST101	*wzi*	*wzi-3*	85	85	84.5	84.8
KP38-19	ST101	*wzi*	*wzi-4*	83.5	83.5	83.5	83.5
KP38-19	ST101	*wzi*	*wzi-3*	85	85	84.5	84.8
KP15-19	ST307	*wzi*	*wzi-4*	83	83	82.5	82.8

Strain name	MLST profile	Gene name	Primer pair name	Melting T 1 (°C)	Melting T 2 (°C)	Melting T 3 (°C)	Average melting T (°C)
KP35-19	ST307	*wzi*	*wzi-4*	83	83	82.5	82.8
KP40-19	ST307	*wzi*	*wzi-3*	84.5	84.5	84.5	84.5
KP40-19	ST307	*wzi*	*wzi-4*	82.5	82.5	82.5	82.5
KP41-19	ST307	*wzi*	*wzi-4*	83	82.5	82.5	82.7
KP41-19	ST307	*wzi*	*wzi-3*	84.5	84.5	84	84.3
KP43-19	ST101	*wzi*	*wzi-3*	85	85	85	85
KP43-19	ST101	*wzi*	*wzi-4*	83.5	83.5	83.5	83.5
KP44-19	ST307	*wzi*	*wzi-4*	82.5	82.5	82.5	82.5
KP44-19	ST307	*wzi*	*wzi-3*	84.5	84.5	84	84.3
KP46-19	ST307	*wzi*	*wzi-3*	84.5	84.5	84	84.3
KP46-19	ST307	*wzi*	*wzi-4*	82.5	82.5	82.5	82.5
KP488	ST258	*wzi*	*wzi-3*	84	84	83.5	83.8
KP488	ST258	*wzi*	*wzi-4*	83.5	83.5	83.5	83.5
KP49-19	ST512	*wzi*	*wzi-3*	83.5	83.5	83.5	83.5
KP49-19	ST512	*wzi*	*wzi-4*	83.5	83.5	83.5	83.5
KP5-19	ST307	*wzi*	*wzi-3*	84	84	84	84
KP5-19	ST307	*wzi*	*wzi-4*	82.5	82.5	82.5	82.5
KP51-19	ST11	*wzi*	*wzi-4*	83.5	83.5	83.5	83.5
KP51-19	ST11	*wzi*	*wzi-3*	85	85	85	85
KP54-19	ST258	*wzi*	*wzi-4*	84.5	84.5	84.5	84.5
KP54-19	ST258	*wzi*	*wzi-3*	83.5	83.5	83.5	83.5
KP56-19	ST512	*wzi*	*wzi-3*	83.5	83.5	83.5	83.5
KP56-19	ST512	*wzi*	*wzi-4*	83.5	83.5	83.5	83.5
KP57-19	ST512	*wzi*	*wzi-3*	83.5	83.5	83.5	83.5
KP57-19	ST512	*wzi*	*wzi-4*	83.5	83.5	83.5	83.5
KP58-19	ST101	*wzi*	*wzi-3*	85	85	85	85
KP58-19	ST101	*wzi*	*wzi-4*	83.5	83.5	83.5	83.5
KP59-19	ST512	*wzi*	*wzi-4*	83.5	83.5	83	83.3
KP59-19	ST512	*wzi*	*wzi-3*	83.5	83.5	83.5	83.5
KP6-19	ST307	*wzi*	*wzi-3*	84	84	84	84
KP6-19	ST307	*wzi*	*wzi-4*	82.5	82.5	82.5	82.5
KP37-19	ST307	*wzi*	*wzi-4*	83	83	82.5	82.8
KP4-19	ST101	*wzi*	*wzi-4*	83.5	83.5	83	83.3
KP7-19	ST307	*wzi*	*wzi-4*	82.5	82.5	82	82.3
KP7-19	ST307	*wzi*	*wzi-3*	84	84	84	84

Strain name	MLST profile	Gene name	Primer pair name	Melting T 1 (°C)	Melting T 2 (°C)	Melting T 3 (°C)	Average melting T (°C)
KP65-18-3	ST101	*wzi*	*wzi-4*	83.5	83.5	83	83.3
KP8-19	ST101	*wzi*	*wzi-4*	83.5	83.5	83	83.3
KP9-19	ST307	*wzi*	*wzi-4*	82.5	82.5	82.5	82.5
KP9-19	ST307	*wzi*	*wzi-3*	84	84	84	84
KP925	ST11	*wzi*	*wzi-4*	83.5	83.5	83.5	83.5
KP925	ST11	*wzi*	*wzi-3*	85	85	85	85

Table S2. P-values of the Wilcoxon test (with Holm post-hoc correction) comparison of the average temperature distance matrices among the ten primer pairs used in this work.

	infB-1	***mdh-1***	***phoE-1***	***rpoB-1***	***tonB-1***	***tonB-2***	***gapA-1***	***pgi-1***	***wzi-3***
mdh-1	1.00000 (n.s)								
phoE-1	0.04820 (*)	1.00000 (n.s)							
rpoB-1	2.3e08 (****)	0.00079 (***)	0.00097 (***)						
tonB-1	1.00000 (n.s)	1.00000 (n.s)	1.00000 (n.s)	0.00099 (***)					
tonB-2	1.00000 (n.s)	1.00000 (n.s)	1.00000 (n.s)	0.00050 (***)	1.00000 (n.s)				
gapA-1	6.9e06 (****)	0.02344 (*)	0.07846 (n.s)	1.00000 (n.s)	0.03806 (*)	0.02619 (*)			
pgi-1	9.0e16 (****)	9.9e09 (****)	3.6e11 (****)	0.04058 (*)	3.9e09 (****)	4.8e10 (****)	0.00099 (***)		
wzi-3	0.00010 (***)	0.00013 (***)	3.6e06 (****)	1.7e11 (****)	0.00204 (**)	0.00318 (**)	2.8e10 (****)	<2e16 (****)	
wzi-4	0.07276 (n.s)	0.02344 (*)	1.4e06 (****)	4.8e13 (****)	0.01799 (*)	0.01594 (*)	2.3e10 (****)	<2e16 (****)	0.11752 (n.s)

Table S3. Features and references of the 82 *Klebsiella pneumoniae* strains used in this work.

Strain name	Collection	MLST profile	*wzi* allele	K-type	Reference	Sequence Read Archive	Hospital
17PV	Background	ST307	*wzi_173*	KL102	Gaiarsa et al. 2015	ERS480608	San Matteo Hospital (Pavia)
51BG	Background	ST1626	*wzi_417*	KL151	Gaiarsa et al. 2015	ERS480639	Papa Giovanni XXIII hospital (Bergamo)
52BG	Background	ST10	*wzi_95*	KL20	Gaiarsa et al. 2015	ERS480640	Papa Giovanni XXIII hospital (Bergamo)
53BG	Background	ST321	*wzi_131*	KL3	Gaiarsa et al. 2015	ERS480641	Papa Giovanni XXIII hospital (Bergamo)
63BG	Background	ST1627	*wzi_418*	KL48	Gaiarsa et al. 2015	ERS480649	Papa Giovanni XXIII hospital (Bergamo)
15PV	Background	ST976	*wzi_415*	KL54	Gaiarsa et al. 2015	ERS480606	San Matteo Hospital (Pavia)
49BG	Background	ST147	*wzi_56*	KL55	Gaiarsa et al. 2015	ERS480637	Papa Giovanni XXIII hospital (Bergamo)
21PV	Background	ST240	*wzi_321*	KL61	Gaiarsa et al. 2015	ERS480661	San Matteo Hospital (Pavia)

55BG	Background	ST466	*wzi_37*	Undetermined	Gaiarsa et al. 2015	ERS480643	Papa Giovanni XXIII hospital (Bergamo)
KP261	Background	ST258	*wzi_29*	KL106	Gona et al. 2019	SRX6820068	San Raffaele hospital (Milan)
KP123	Background	ST512	*wzi_154*	KL107	Gona et al. 2019	SRX6820115	San Raffaele hospital (Milan)
KP359	Background	ST149	*wzi_62*	KL62	Gona et al. 2019	SRX6820077	San Raffaele hospital (Milan)
KP248	Background	ST423	*wzi_8*	Undetermined	Gona et al. 2019	SRX6820129	San Raffaele hospital (Milan)
KP366	Background	ST11	*wzi_75*	Undetermined	Gona et al. 2019	SRX6820117	San Raffaele hospital (Milan)
KP469	Background	ST395	*wzi_2*	Undetermined	Gona et al. 2019	SRX6820062	San Raffaele hospital (Milan)
KP473	Background	ST101	*wzi_137*	Undetermined	Gona et al. 2019	SRX6820096	San Raffaele hospital (Milan)
KP485	Background	ST15	*wzi_89*	Undetermined	Gona et al. 2019	SRX6820105	San Raffaele hospital (Milan)
BG-Kpn-101-19	Validation	ST15	*wzi_24*	KL24	This work	ERS4238606	Papa Giovanni XXIII hospital (Bergamo)
BG-Kpn-103-19	Validation	ST15	*wzi_24*	KL24	This work	ERS4238607	Papa Giovanni XXIII hospital (Bergamo)
BG-Kpn-20-18	Outbreak	ST512	*wzi_154*	KL107	This work	ERS4238608	Papa Giovanni XXIII hospital (Bergamo)
BG-Kpn-21-18	Outbreak	ST512	*wzi_154*	KL107	This work	ERS4238609	Papa Giovanni XXIII hospital (Bergamo)
BG-Kpn-22-18	Outbreak	ST307	*wzi_173*	KL102	This work	ERS4238610	Papa Giovanni XXIII hospital (Bergamo)
BG-Kpn-23-18	Outbreak	ST512	*wzi_154*	KL107	This work	ERS4238611	Papa Giovanni XXIII hospital (Bergamo)
BG-Kpn-24-18	Outbreak	ST512	*wzi_154*	KL107	This work	ERS4238612	Papa Giovanni XXIII hospital (Bergamo)
BG-Kpn-25-18	Outbreak	ST512	*wzi_154*	KL107	This work	ERS4238613	Papa Giovanni XXIII hospital (Bergamo)
BG-Kpn-26-18	Outbreak	ST512	*wzi_154*	KL107	This work	ERS4238614	Papa Giovanni XXIII hospital (Bergamo)
BG-Kpn-27-18	Outbreak	ST512	*wzi_154*	KL107	This work	ERS4238615	Papa Giovanni XXIII hospital (Bergamo)
BG-Kpn-28-18	Outbreak	ST512	*wzi_154*	KL107	This work	ERS4238616	Papa Giovanni XXIII hospital (Bergamo)
54BG	Validation	ST258	*wzi_29*	KL106	Gaiarsa et al. 2015	ERS480642	Papa Giovanni XXIII hospital (Bergamo)
56BG	Validation	ST258	*wzi_29*	KL106	Gaiarsa et al. 2015	ERS480644	Papa Giovanni XXIII hospital (Bergamo)
57BG	Validation	ST258	*wzi_29*	Undetermined	Gaiarsa et al. 2015	ERS480645	Papa Giovanni XXIII hospital (Bergamo)
KP232	Validation	ST258	*wzi_154*	KL107	Gona et al. 2019	SRX6820131	San Raffaele hospital (Milan)
KP252	Validation	ST258	*wzi_154*	KL107	Gona et al. 2019	SRX6820065	San Raffaele hospital (Milan)
KP488	Validation	ST258	*wzi_154*	KL107	Gona et al. 2019	SRX6820136	San Raffaele hospital (Milan)
KP246	Validation	ST258	*wzi_29*	Undetermined	Gona et al. 2019	SRX6820130	San Raffaele hospital (Milan)
BG-Kpn-29-18	Outbreak	ST512	*wzi_154*	KL107	This work	ERS4238617	Papa Giovanni XXIII hospital (Bergamo)
BG-Kpn-30-18	Outbreak	ST512	*wzi_154*	KL107	This work	ERS4238618	Papa Giovanni XXIII hospital (Bergamo)
KP1-19	Validation	ST101	*wzi_137*	KL17	This work	ERS4238621	San Raffaele hospital (Milan)
KP108-18-2	Validation	ST512	*wzi_154*	KL107	This work	ERS4238619	San Raffaele hospital (Milan)

KP11-19	Validation	ST512	*wzi_154*	KL107	This work	ERS4238620	San Raffaele hospital (Milan)
KP12-19	Validation	ST512	*wzi_154*	KL107	This work	ERS4238623	San Raffaele hospital (Milan)
KP12-19-2	Validation	ST512	*wzi_154*	KL107	This work	ERS4238622	San Raffaele hospital (Milan)
KP13-19	Validation	ST307	*wzi_173*	KL102	This work	ERS4238624	San Raffaele hospital (Milan)
KP14-19	Validation	ST512	*wzi_154*	KL107	This work	ERS4238625	San Raffaele hospital (Milan)
KP15-19	Validation	ST307	*wzi_173*	KL102	This work	ERS4238626	San Raffaele hospital (Milan)
KP17-19	Validation	ST101	*wzi_137*	KL17	This work	ERS4238627	San Raffaele hospital (Milan)
KP18-19	Validation	ST101	*wzi_137*	KL17	This work	ERS4238628	San Raffaele hospital (Milan)
KP19-19	Validation	ST512	*wzi_154*	KL107	This work	ERS4238629	San Raffaele hospital (Milan)
KP2-19	Validation	ST307	*wzi_173*	KL102	This work	ERS4238632	San Raffaele hospital (Milan)
KP20-19	Validation	ST258	*wzi_29*	KL106	This work	ERS4238630	San Raffaele hospital (Milan)
KP21-19	Validation	ST258	*wzi_29*	Undetermined	This work	ERS4238631	San Raffaele hospital (Milan)
KP24-19	Validation	ST512	*wzi_154*	KL107	This work	ERS4238633	San Raffaele hospital (Milan)
KP25-19	Validation	ST307	*wzi_173*	Undetermined	This work	ERS4238634	San Raffaele hospital (Milan)
KP26-19	Validation	ST307	*wzi_173*	KL102	This work	ERS4238635	San Raffaele hospital (Milan)
KP27-19	Validation	ST307	*wzi_173*	KL102	This work	ERS4238636	San Raffaele hospital (Milan)
KP29-19	Validation	ST512	*wzi_154*	KL107	This work	ERS4238637	San Raffaele hospital (Milan)
KP3-19	Validation	ST307	*wzi_173*	KL102	This work	ERS4238638	San Raffaele hospital (Milan)
KP34-19	Validation	ST101	*wzi_137*	KL17	This work	ERS4238639	San Raffaele hospital (Milan)
KP35-19	Validation	ST307	*wzi_173*	KL102	This work	ERS4238640	San Raffaele hospital (Milan)
KP36-19	Validation	ST101	*wzi_137*	KL17	This work	ERS4238641	San Raffaele hospital (Milan)
KP37-19	Validation	ST307	*wzi_173*	KL102	This work	ERS4238642	San Raffaele hospital (Milan)
KP38-19	Validation	ST101	*wzi_137*	KL17	This work	ERS4238643	San Raffaele hospital (Milan)
KP4-19	Validation	ST101	*wzi_137*	Undetermined	This work	ERS4238646	San Raffaele hospital (Milan)
KP40-19	Validation	ST307	*wzi_173*	KL102	This work	ERS4238644	San Raffaele hospital (Milan)
KP41-19	Validation	ST307	*wzi_173*	KL102	This work	ERS4238645	San Raffaele hospital (Milan)
KP43-19	Validation	ST101	*wzi_137*	KL17	This work	ERS4238647	San Raffaele hospital (Milan)
KP44-19	Validation	ST307	*wzi_173*	KL102	This work	ERS4238648	San Raffaele hospital (Milan)
KP46-19	Validation	ST307	*wzi_173*	Undetermined	This work	ERS4238649	San Raffaele hospital (Milan)
KP49-19	Validation	ST512	*wzi_154*	KL107	This work	ERS4238650	San Raffaele hospital (Milan)
KP5-19	Validation	ST307	*wzi_173*	KL102	This work	ERS4238652	San Raffaele hospital (Milan)

KP51-19	Validation	ST11	*wzi_24*	KL24	This work	ERS4238651	San Raffaele hospital (Milan)
KP54-19	Validation	ST258	*wzi_29*	Undetermined	This work	ERS4238653	San Raffaele hospital (Milan)
KP56-19	Validation	ST512	*wzi_154*	KL107	This work	ERS4238654	San Raffaele hospital (Milan)
KP57-19	Validation	ST512	*wzi_154*	KL107	This work	ERS4238655	San Raffaele hospital (Milan)
KP58-19	Validation	ST101	*wzi_137*	Undetermined	This work	ERS4238656	San Raffaele hospital (Milan)
KP59-19	Validation	ST512	*wzi_154*	KL107	This work	ERS4238657	San Raffaele hospital (Milan)
KP6-19	Validation	ST307	*wzi_173*	Undetermined	This work	ERS4238658	San Raffaele hospital (Milan)
KP65-18-3	Validation	ST101	*wzi_137*	KL17	This work	ERS4238659	San Raffaele hospital (Milan)
KP7-19	Validation	ST307	*wzi_173*	Undetermined	This work	ERS4238660	San Raffaele hospital (Milan)
KP8-19	Validation	ST101	*wzi_137*	Undetermined	This work	ERS4238661	San Raffaele hospital (Milan)
KP9-19	Validation	ST307	*wzi_173*	Undetermined	This work	ERS4238662	San Raffaele hospital (Milan)
KP925	Validation	ST11	*wzi_24*	KL24	This work	ERS4238663	San Raffaele hospital (Milan)

Table S4. Clinical information of the outbreak strains.

Strain name	Isolation date (yyyy/mm/dd)	Ward	Material
BG-Kpn-20-18	2018/04/10	Infectious Diseases	Rectal swab
BG-Kpn-21-18	2018/04/10	Infectious Diseases	Rectal swab
BG-Kpn-22-18	2018/04/11	Oncology	Rectal swab
BG-Kpn-23-18	2018/04/15	Oncology	Rectal swab
BG-Kpn-24-18	2018/04/16	Infectious Diseases	Pus
BG-Kpn-25-18	2018/04/17	Oncology	Rectal swab
BG-Kpn-26-18	2018/04/20	Cardio-Surgical Intensive Care unit	Blood
BG-Kpn-27-18	2018/04/24	Infectious Diseases	Rectal swab
BG-Kpn-28-18	2018/04/24	Infectious Diseases	Urine
BG-Kpn-29-18	2018/04/24	Oncology	Rectal swab
BG-Kpn-30-18	2018/04/26	Cardio-Surgical Intensive Care unit	Rectal swab

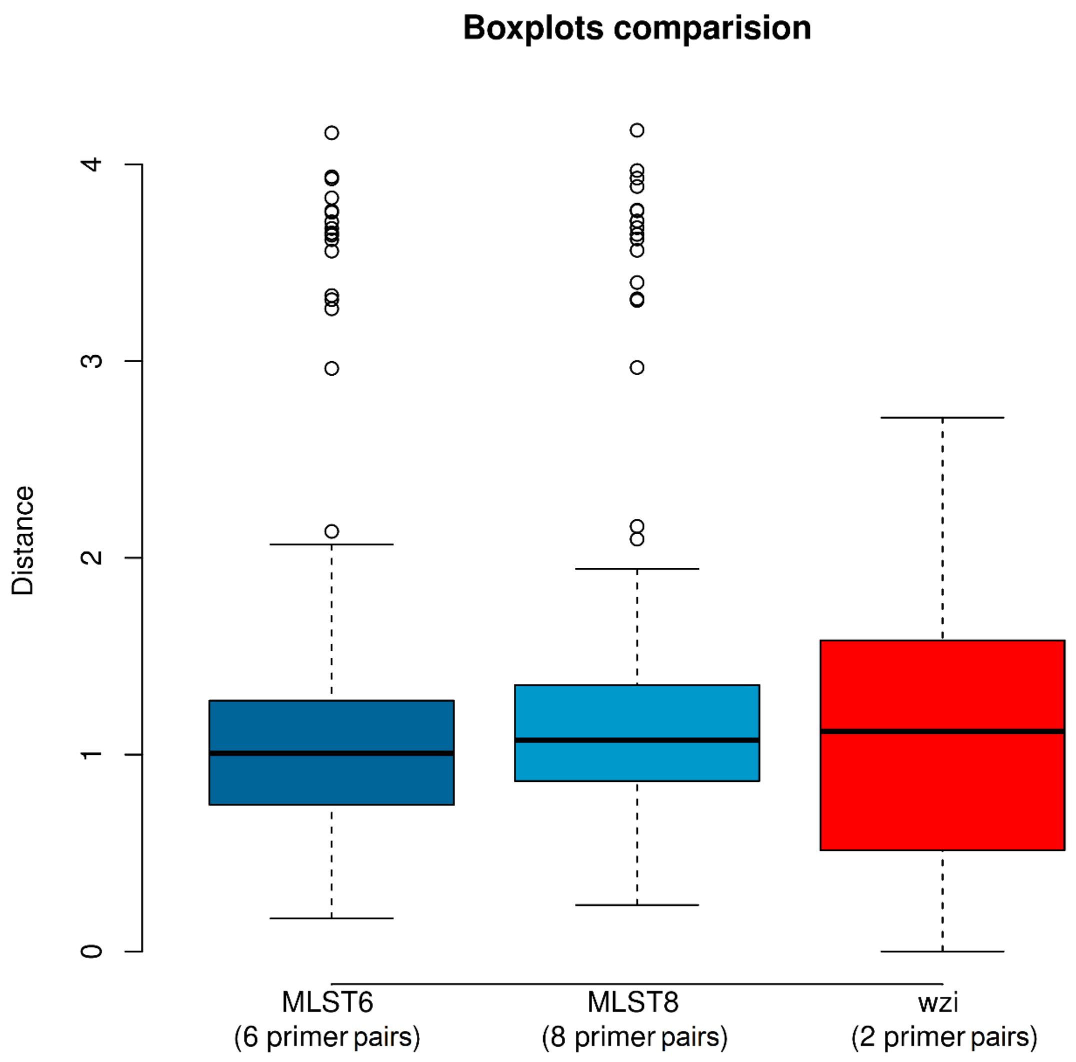

Figure S3. Distribution of the average melting temperature difference among the 17 *K. pneumoniae* strains for the three primer schemes. Boxes are the 25th and 75th quartiles divided by the medians, whiskers are 1.5x the interquartile ranges and dots are outliers.

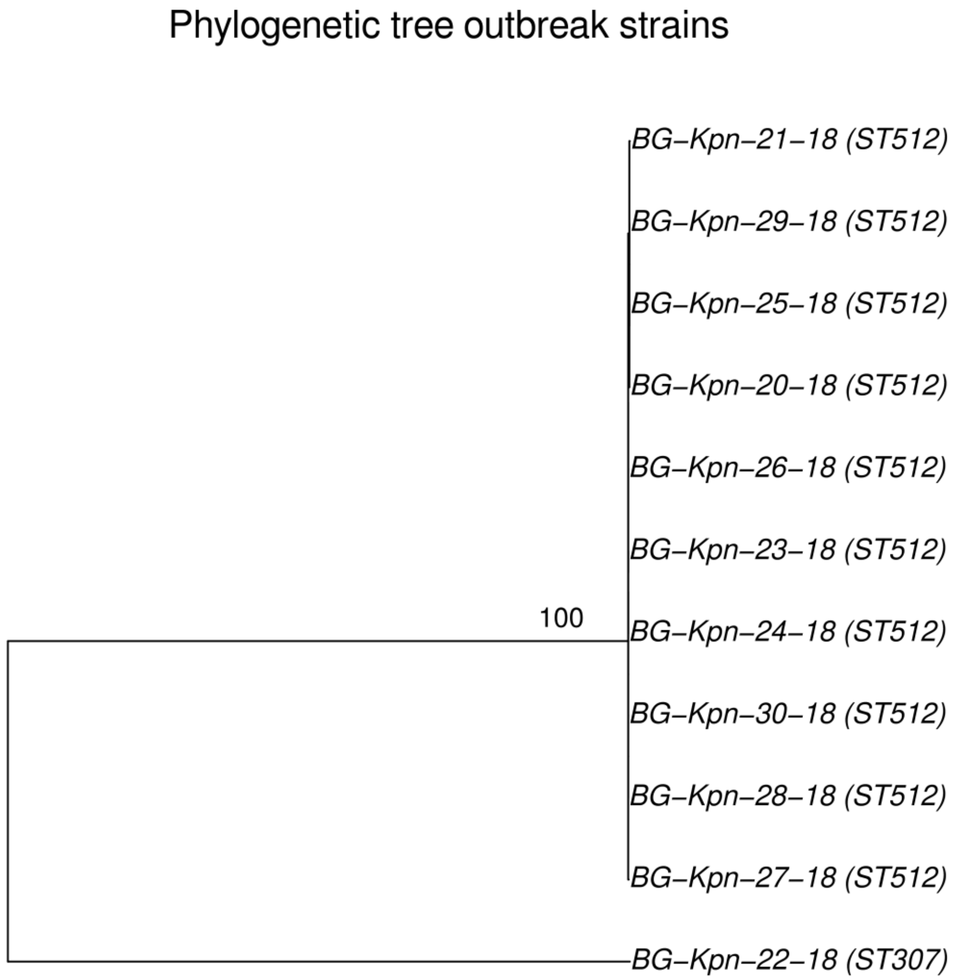

Figure S4. Maximum Likelihood phylogenetic tree of the 11 outbreak strains. The tree was obtained from an alignment of 66 core-SNPs

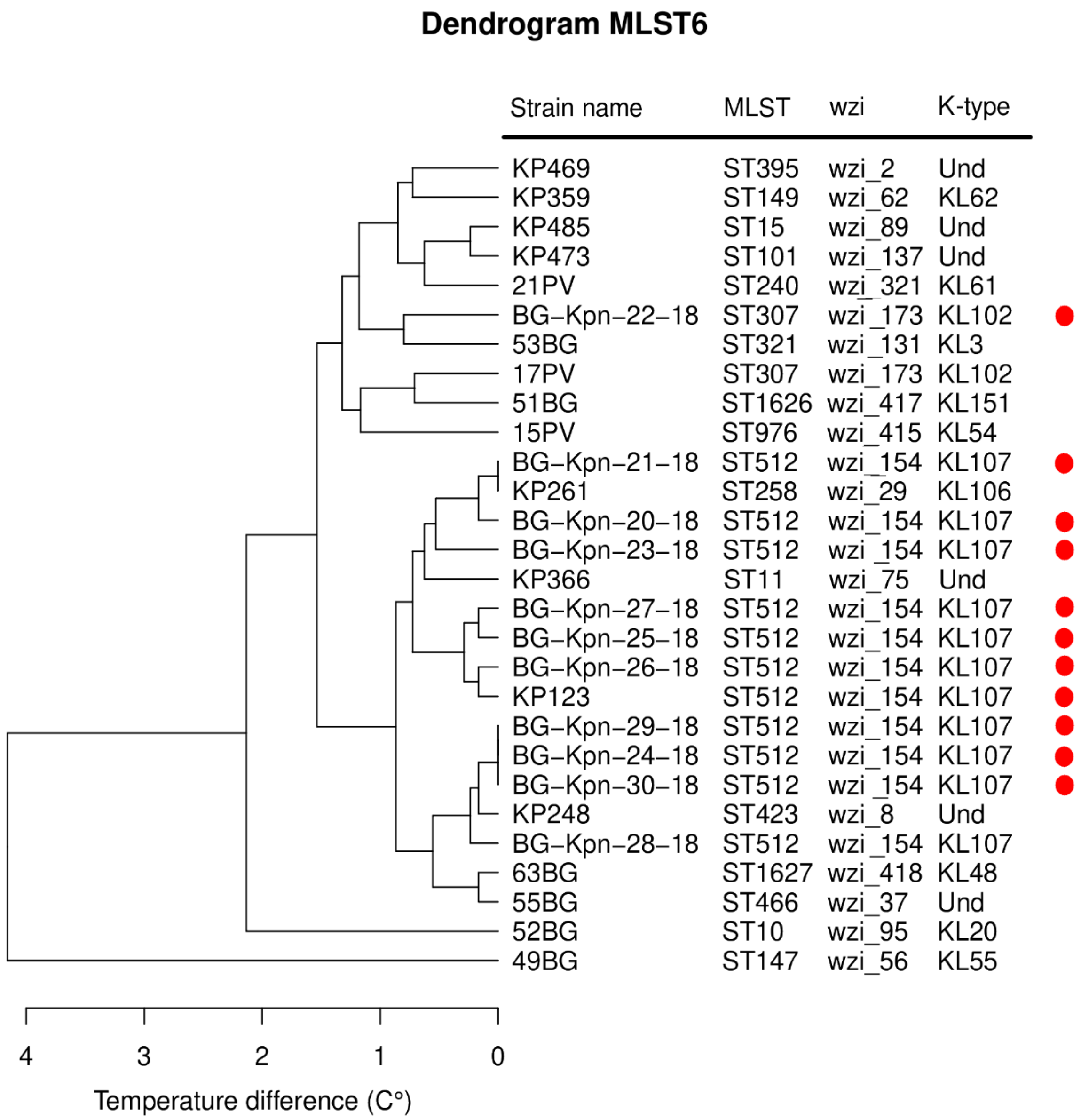

Figure S5. Dendrogram of the hierarchical clustering analysis on the average temperature distance matrix obtained using the MLST6 scheme. The 17 “background” strains belonging to 17 different MLSTs are written in black, while the 11 strains isolated during the nosocomial outbreak are highlighted by a red dot.

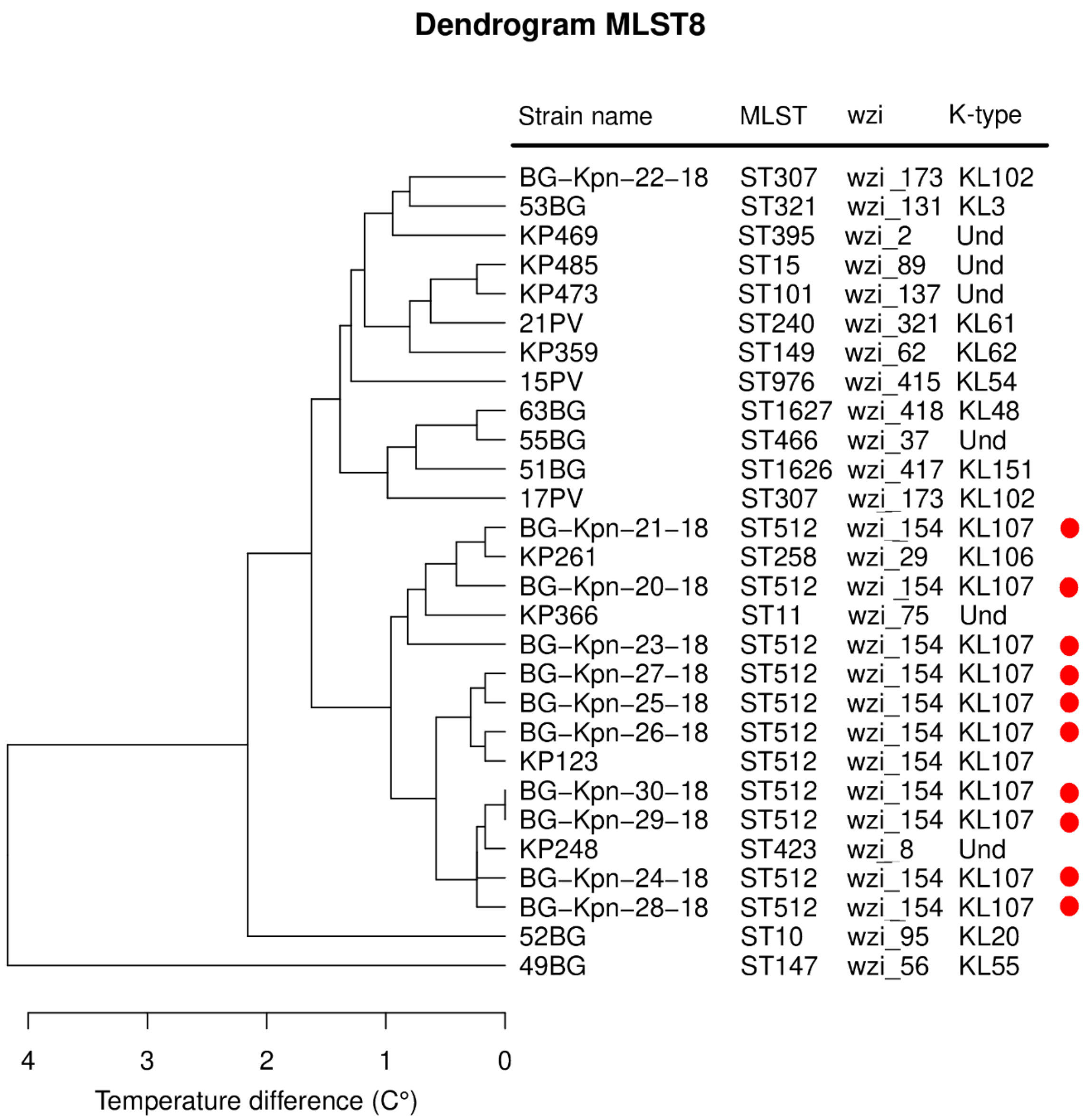

Figure S6. Dendrogram of the hierarchical clustering analysis on the average temperature distance matrix obtained using the MLST8 scheme. The 17 "background" strains belonging to 17 different MLSTs are written in black, while the 11 strains isolated during the nosocomial outbreak are highlighted by a red dot.

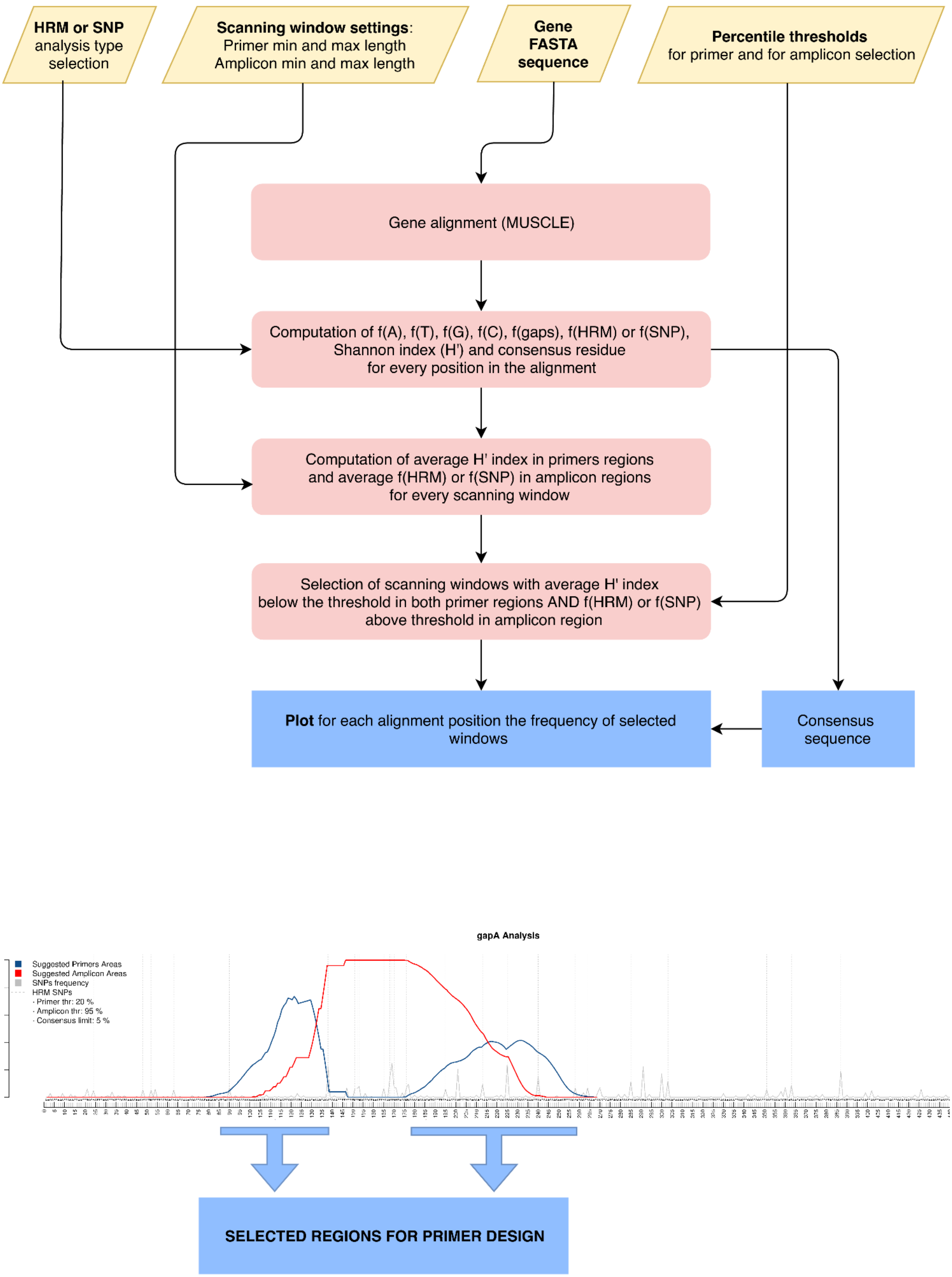

Figure S7. The diagram summarizes the main steps of the algorithm of EasyPrimer and shows an example of output
